# Yin Yang 1 facilitates hepatocellular carcinoma cell lipid metabolism and tumor progression by inhibiting PGC-1β-induced fatty acid oxidation

**DOI:** 10.7150/thno.34931

**Published:** 2019-10-14

**Authors:** Yanjun Li, Vivi Kasim, Xuesong Yan, Lang Li, Ian Timothy Sembiring Meliala, Can Huang, Zhuolin Li, Ke Lei, Guanbin Song, Xiaodong Zheng, Shourong Wu

**Affiliations:** 1 The Key Laboratory of Biorheological Science and Technology, Ministry of Education, College of Bioengineering, Chongqing University, Chongqing 400044, China.; 2 The 111 Project Laboratory of Biomechanics and Tissue Repair, College of Bioengineering, Chongqing University, Chongqing 400044, China.; 3 State and Local Joint Engineering Laboratory for Vascular Implants, Chongqing University, Chongqing 400044, China.; 4 Chongqing University Cancer Hospital, Chongqing University, Chongqing 400030, China.

**Keywords:** Yin Yang 1, fatty acid oxidation, lipid accumulation, hepatocellular carcinoma, PGC-1β

## Abstract

Lipid accumulation is a driving force in tumor development, as it provides tumor cells with both energy and the building blocks of phospholipids for construction of cell membranes. Aberrant homeostasis of lipid metabolism has been observed in various tumors; however, the molecular mechanism has not been fully elucidated.

**Methods:** Yin yang 1 (YY1) expression in hepatocellular carcinoma (HCC) was analyzed using clinical specimens, and its roles in HCC in lipid metabolism were examined using gain- and loss-of function experiments. The mechanism of YY1 regulation on peroxisome proliferator-activated receptor gamma coactivator-1β (PGC-1β) and its downstream genes medium-chain acyl-CoA dehydrogenase (MCAD) and long-chain acyl-CoA dehydrogenase (LCAD) were investigated using molecular biology and biochemical methods. The role of YY1/ PGC-1β axis in hepatocarcinogenesis was studied using xenograft experiment.

**Results:** This study showed that YY1 suppresses fatty acid β-oxidation, leading to increase of cellular triglyceride level and lipid accumulation in HCC cells, and subsequently induction of the tumorigenesis potential of HCC cells. Molecular mechanistic study revealed that YY1 blocks the expression of *PGC-1β*, an activator of fatty acid β-oxidation, by directly binding to its promoter; and thus downregulates PGC-1β/MCAD and PGC1-β/LCAD axis. Importantly, we revealed that YY1 inhibition on *PGC-1β* occurs irrespective of the expression of hypoxia-inducible factor-1α (HIF1-α), enabling it to promote lipid accumulation under both normoxic and hypoxic conditions.

**Conclusion:** Our study reveals the critical role of YY1/PGC-1β axis in HCC cell lipid metabolism, providing novel insight into the molecular mechanisms associated with tumor cell lipid metabolism, and a new perspective regarding the function of YY1 in tumor progression. Thus, our study provides evidences regarding the potential of YY1 as a target for lipid metabolism-based anti-tumor therapy.

## Introduction

Metabolic reprogramming is a characteristic of tumor cells and is closely related to malignancy 1, 2. Recent studies revealed alterations in lipid metabolism as an important hallmark of tumor metabolic reprogramming 3. Fatty acids are not only important sources of cell energy but also major sources for cell-membrane synthesis and signaling molecules 4, 5. In normal cells, the balance of cellular fatty acid content is determined by the balance of fatty acid synthesis (FAS) and degradation maintained by fatty acid oxidation (FAO) pathways 6. However, to meet the demand of their highly proliferative growth, tumor cells alter their lipid metabolism by accelerating *de novo* fatty acid synthesis while simultaneously suppressing fatty acid degradation, resulting in cellular lipid accumulation. Furthermore, tumor microenvironment, such as hypoxia, is also an important driving force for tumor cell lipid metabolic reprogramming 7-9. On the other hand, recent studies demonstrated that disrupted lipid homeostasis is also highly related with the most common chronic liver disease, nonalcoholic fatty liver disease (NAFLD), whose full spectrum ranges from isolated hepatic steatosis to nonalcoholic steatohepatitis (NASH). NASH could progress to cirrhosis, which in turn predisposes patients to hepatocellular carcinoma (HCC) 10, 11.

Yin yang 1 ( YY1 ) is a GLI-Krüppel zinc finger protein with four C2H2 zinc finger domains at its carboxy terminus which could act as both positive and negative regulators of target genes depending on the contexts 12, 13. YY1 could bind and regulate its target genes both at their transcriptional 14-16 and post-translational 17-19 levels. Furthermore, recent study reveals that YY1 could also control gene expression by binding to active enhancers and promoter-proximal elements, facilitating the interaction of these DNA elements 20. YY1 plays critical roles in various biological processes, including DNA replication, cell proliferation and differentiation, and embryonic development 13, 19, 21, 22. Aberrant YY1 expression is closely related to diseases, and its overexpression is observed in various cancers including HCC 23-27. A previous study showed that YY1 suppresses *C/EBP homologous protein* transcription and induces the accumulation of triglyceride (TG) in adipocytes, suggesting its relationship with obesity 28. Furthermore, YY1 increases in the liver of obese mice and promotes cellular TG accumulation in adipocytes by suppressing the expression of the *farnesoid X receptor* gene, thereby leading to increased hepatosteatosis 29. These studies indicate that YY1 might be involved in lipid metabolic disorder diseases; however, YY1 involvement in altering tumor cell lipid metabolism has not been fully elucidated.

Here, we revealed that YY1 is critical for alteration of lipid metabolism in HCC cells by suppressing the expression of *peroxisome proliferator-activated receptor gamma coactivator-1β* (*PGC-1β*), a transcriptional activator of *medium-chain acyl-CoA dehydrogenase* (*MCAD*) and *long-chain acyl-CoA dehydrogenase* (*LCAD*) 7, 30. PGC-1β enhances the expression levels of both MCAD and LCAD, which are key enzymes necessary for FAO, and thus are critical for promoting tumorigenesis 7, 31. In line with this, our findings demonstrated that YY1 overexpression suppresses fatty acid β-oxidation, leading to increased lipid accumulation in HCC cells and subsequent hepatocarcinogenesis. Interestingly, our results showed that YY1 inhibition of *PGC-1β* expression occurs independent of hypoxia-inducible factor-1α (HIF-1α), a key regulator of hypoxic response that has been known to promote tumor cell lipid accumulation. Accordingly, YY1 could alter HCC cell lipid metabolism under both normoxic and hypoxic conditions. These results provide novel insight into the molecular mechanisms associated with cell lipid metabolic reprogramming, an important hallmark and driving force of hepatocarcinogenesis.

## Methods

### Vectors construction

U6 promoter-based shRNA expression vectors specific for *YY1, MCAD, LCAD, PGC-1β* and *HIF-1α* were designed and constructed as described previously 32. Target sequences were as follows: shYY1-1 (5'-GCA AGA AGA GTT ACC TCA G-3'); shYY1-2 (5'-GGC AGA ATT TGC TAG AAT G-3'); shMCAD-1 (5'-GCA CCA AGC AAT ATC ATT T-3'); shMCAD-2 (5'-GGA GAA AGG AAT TAA ACA T-3'); shLCAD-1 (5'-GGT AAG AAG TAA ATA TGT A-3'); shLCAD-2 (5'-GAA AGA GCT TCC ACA GGA A-3'); shPGC-1β-1 (5'-TGA GTA TGA CAC TGT CTT T-3'); shPGC-1β-2 (5'-CAG ATA CAC TGA CTA CGA T-3'); shHIF-1α-1 (5'-GGA TGA AAG TGG ATT ACC A-3') and shHIF-1α-2 (5'-GAC ACA GCC TGG ATA TGA A-3'). shRNA expression vector containing a stretch of 7 thymines terminator sequences exactly downstream of the U6 promoter, namely shCon, was used as a control. *YY1* (pcYY1), *HIF-1α* (pcHIF-1α), and *YY2* (pcYY2) overexpression vectors were constructed as described previously 19, 24. For *YY1* and *PGC-1β* overexpression vectors with puromycin resistance gene (pcEF9-puro-YY1 and pcEF9-puro-PGC-1β), the corresponding coding sequences were further subcloned into pcEF9-puro vector (kindly provided by Dr. Makoto Miyagishi, AIST, Japan).

For wild-type PGC-1β luciferase reporter vector (PGC-1β-Luc) and PGC-1β luciferase reporter vector without predicted YY1 binding site (PGC-1βdel-Luc), we cloned the -594 to +532 and the -594 to -226 regions of the *PGC-1β* promoter, respectively, into the *Bgl*II and *Hind*III sites of the pGL4.13 vector (Promega, Madison, WI). Human genome DNA was extracted from HepG2 cells using TIANamp Genomic DNA Kit (Tiangen Biotech, Beijing, China), and used as template. Promoter regions were then amplified using Takara PrimeSTAR Max DNA Polymerase (Takara Bio, Dalian, China). PGC-1β luciferase reporter vector with mutated YY1 binding site (PGC-1β^Mut^-Luc) were constructed using Site-directed mutagenesis kit (Beyotime, Shanghai, China).

### Cell lines, cell culture and transient transfection

HepG2 and MHCC-97H cell lines were purchased from the Cell Bank of Chinese Academy of Sciences (Shanghai, China), and cultured in Dulbecco's modified Eagle's medium (Gibco, Life Technologies, Grand Island, NY) supplemented with 10% fetal bovine serum (Biological Industries, Beit Haemek, Israel) and 1% penicillin-streptomycin. Cell lines were verified using short-tandem repeat profiling method, and were tested periodically for mycoplasma contamination by using Mycoplasma Detection Kit-Quick Test (Biotool, Houston, TX).

For gene-silencing experiments, cells were seeded in 6-well plate and transfected with 2 μg of indicated vectors. 24 h after transfection, transfected cells were selected by using 1 μg/ml puromycin for 36 h. For gene overexpression experiments, cells were seeded in 6 well-plates, transfected with 2 μg of indicated vectors, and collected 24 h after transfection for further experiments. For double silencing and triple silencing experiments, cells were transfected with 1 μg or 0.7 μg of each indicated vectors respectively, and subjected to puromycin selection to eliminate untransfected cells. For establishing *YY1-*silenced and *YY1*/*PGC-1β*-double silenced MHCC-97H stable cell lines, cells were seeded in 10 cm well-plates, and transfected with 7.5 μg each of shYY1-1 and shCon vectors, or 7.5 μg each of shYY1 and shPGC-1β-1 vectors. Cells transfected with 15 μg shCon were used as control. For establishing *YY1*-overexpressed and *YY1/PGC-1β-*double overexpressed MHCC-97H stable cell lines, cells were seeded in 10 cm well-plates, and transfected with 7.5 μg of pcEF9-puro-YY1, or 7.5 μg each of pcEF9-puro-YY1 and pcEF9-puro-PGC-1β vectors. Cells transfected with 15 μg pcEF9-puro were used as control. Stable cell lines were then established by performing puromycin selection. HIF-1α-null HepG2 (HepG2^HIFnull^) stable cells were established using CRISPR/Cas9 method. Briefly, cells were transfected with vectors targeting *HIF-1α* (GeneCopoiea, Rockville, MD; HCP001130-CG09-3-10-a, target site: TTC TTT ACT TCG CCG AGA TC; HCP001130-CG09-3-10-b, target site: CCA TCA GCT ATT TGC GTG TG; HCP001130-CG09-3-10-c, target site: TGT GAG TTC GCA TCT TGA TA). Twenty-four hours later, puromycin selection (1 μg/ml) was performed for 7 days to eliminate untransfected cells. Cell line was then established from a single clone. The corresponding genome DNA was subjected to sequencing and deletion of nucleotides located in 567 to 606 region (40 bp) of *HIF-1α* coding sequence was confirmed. All transfections were performed using Lipofectamine 2000 (Invitrogen Life Technologies) according to the manufacturer's instruction. Hypoxia treatment was performed as described in our previous work for 24 h or 48 h before RNA or protein extraction, respectively 19.

### Clinical human HCC specimens

Human HCC specimens were obtained from patients undergoing surgery at Chongqing University Cancer Hospital (Chongqing, China). Patients did not receive chemotherapy, radiotherapy or other adjuvant therapies prior to the surgery. The specimens were snap-frozen in liquid nitrogen. Prior patients' written informed consents were obtained. The experiments were approved by the Institutional Research Ethics Committee of Chongqing University Cancer Hospital, and conducted in accordance with Declaration of Helsinki.

### Xenograft experiment

For the* in vivo* tumor study, BALB/c-nu/nu mice (male, body weight: 18-22 g, 6 weeks old) were purchased from the Third Military Medical University (Chongqing, China). Animal studies were carried out in the Third Military Medical University (Chongqing, China, Permit Number SYXK-PLA-20120031), and approved by the Laboratory Animal Welfare and Ethics Committee of the Third Military Medical University. All animal experiments conformed to the approved guidelines of Animal Care and Use Committee of Third Military Medical University. All efforts were made to minimize suffering.

For generating experimental subcutaneous tumor model, BALB/c-nu/nu mice were randomly divided into three groups (n = 6), and each group was injected subcutaneously with stable cell lines. Tumor size (V) was evaluated by caliper every four days with reference to the following equation: V = a x b^2^/2, where a and b are the major and minor axes of the tumor, respectively 19. The investigator was blinded to the group allocation and during the assessment.

### RNA extraction, quantitative reverse-transcribed polymerase chain reaction (qRT-PCR) analysis and western blotting

Detailed methods for RNA extraction, qRT-PCR and western blotting are described in the Supplementary Materials and Methods. The sequences of the primers and the antibodies used are listed in **Table S1** and **Table S2**, respectively.

### Fatty acid β-oxidation rate

Cells were transfected with indicated shRNA expression vectors or overexpression vectors as described above. Cells or tissues were collected and mitochondrial protein was extracted using Cell Mitochondria Isolation Kit (Solarbio, Beijing, China). Fatty acid β-oxidation rate was measured using Fatty Acid β-Oxidation Detection Kit (Genmed Scientifics, Shanghai, China). Values were normalized with the amount of mitochondrial protein.

### Statistical analysis

All values were presented as mean ± SEM from three independent experiments. Quantification results were analyzed as a relative to that of controls of each experiment, which were defined as one, and then presented as an average of three independent experiments. Statistical analysis was performed by One-way ANOVA conducted using SPSS Statistics v. 17.0. Statistical significance was defined as *P* < 0.05, and *P* < 0.01 was considered highly significant compared to control group. For analyzing the correlation of two variables in human HCC specimens, bivariate correlation analysis (Pearson's r test) was conducted using Prism5.

## Results

### YY1 enhances lipid accumulation in HCC cells

**Figure 1:**
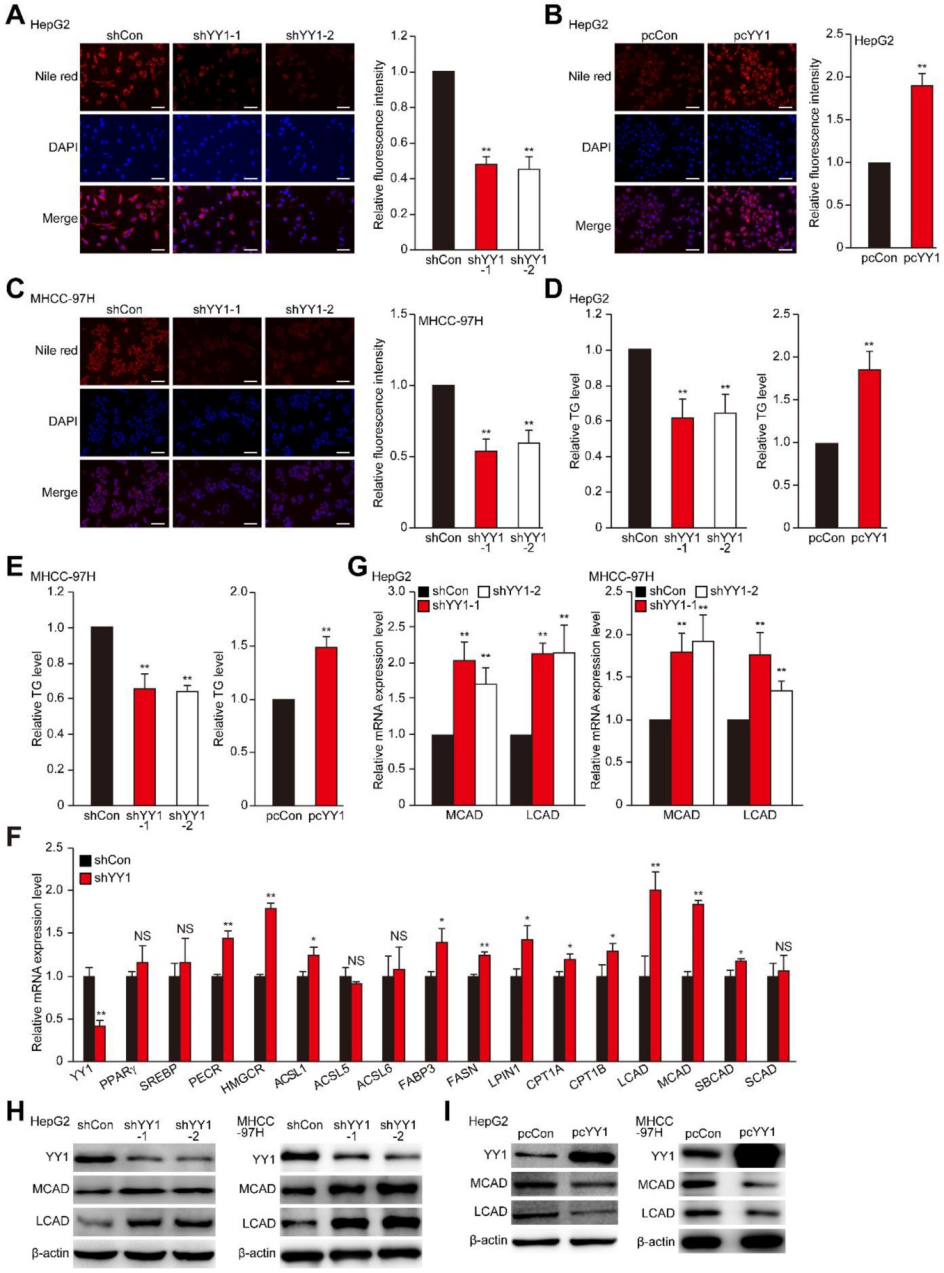
** YY1 induces hepatocellular carcinoma (HCC) cell lipid accumulation by regulating MCAD and LCAD expression. A-B.** The accumulation of lipid droplets in *YY1*-silenced (A) and *YY1*-overexpressed (B) HepG2 cells, as determined using Nile Red staining. Representative images (left) and relative fluorescence intensity (right, n = 9) are shown. **C.** The accumulation of lipid droplets in *YY1*-silenced MHCC-97H cells, as examined using Nile Red staining. Representative images (left) and relative fluorescence intensity (right, n = 9) are shown.** D-E.** The levels of cellular triglyceride (TG) in *YY1*-silenced and *YY1*-overexpressed HepG2 (D) and MHCC-97H (E) cells (n = 3). **F.** mRNA expression levels of various lipid metabolic-associated factors in *YY1*-silenced HepG2 cells, as determined using quantitative reverse-transcribed PCR (qRT-PCR). Representative data are shown (n = 3). **G-H.** mRNA (G) and protein (H) expression levels of MCAD and LCAD in *YY1*-silenced HepG2 and MHCC-97H cells, as determined using qRT-PCR (n = 3) and western blotting, respectively. **I.** Protein expression levels of MCAD and LCAD in *YY1*-overexpressed HepG2 and MHCC-97H cells, as determined using western blotting. All experiments were performed under hypoxic condition. Cells transfected with shCon or pcCon were used as controls. β-actin was used for qRT-PCR normalization and as western blotting loading control. Scale bars: 200 μm. Quantification data are shown as mean ± SEM of three independent experiments. pcCon: pcDNA3.1(+); *P < 0.05; **P < 0.01; NS: not significant (ANOVA).

Accumulation of lipid is crucial for tumor cells to meet the demand of their highly proliferative growth. Hypoxia is an important driving force for tumor cell lipid accumulation 8, 9. As shown in **Figure S1A**, hypoxic environment enhances the level of TG, which in turn contributes to tumor cell metabolic plasticity and promotes tumor cell growth, as well as metastasis 33, 34. To investigate the role of YY1 in regulating HCC cell lipid metabolism under hypoxic condition, we knocked down *YY1* expression in the HCC cell lines HepG2 and MHCC-97H using short-hairpin RNAs (shRNAs), and confirmed that both shYY1 vectors efficiently knocked down YY1 mRNA expression (**Figure S1B**). To exclude the cross-reactivity of the anti-YY1 antibody we used with YY2, another member of YY1 family highly homologous with YY1, we examined the specificity of anti-YY1 antibody in *YY2*-overexpressed HepG2 cells. As shown in **Figure S1C**, anti-YY1 antibody could only detect the band of YY1 but not that of YY2 both in control and in *YY2*-overexpressed cells, confirming that the antibody we used was specific to YY1. Using this antibody, we further confirmed the efficiency of shYY1 in downregulating YY1 protein expression (**Figure S1D**). We next examined alterations in lipid accumulation using Nile Red and Oil Red O stainings, and found that *YY1* silencing significantly suppressed lipid accumulation in HepG2 cells under hypoxic condition (**Figure 1A, Figure S1E**). Concomitantly, *YY1* overexpression (**Figure S1F**) enhanced lipid accumulation (**Figure 1B**). Similar tendency was also observed in MHCC-97H cells (**Figure 1C**). Evaluation of changes in TG level also revealed that under hypoxic condition, *YY1* silencing significantly suppressed TG level in both cell lines, whereas* YY1* overexpression promoted it (**Figure 1D-E**). These results suggested that YY1 is critical for lipid accumulation in HCC cells.

To uncover the mechanisms underlying YY1 regulation of HCC cell lipid metabolism, we investigated the effect of *YY1* silencing and overexpression on the expression of genes associated with lipid metabolism in tumors 4, 7. As shown in **Figure 1F**, among the genes whose expression levels were affected by *YY1*-silencing, the levels of medium-chain acyl-CoA dehydrogenase (MCAD), long-chain acyl-CoA dehydrogenase (LCAD) and 3-hydroxy-3-methylglutaryl-CoA-reductase (HMGCR) were induced most significantly. In line with the results of *YY1* silencing, *YY1* overexpression significantly suppressed MCAD and LCAD expression; however, it did not significantly affect HMGCR expression (**Figure S2A**). Previous studies showed that MCAD and LCAD are key enzymes involved in fatty acid β-oxidation and, therefore, are critical for fatty acid degradation 7, 31; while HMGCR is the rate limiting enzyme in cholesterol biosynthesis whose upregulation promotes lipid accumulation 35. Given that *YY1*-silencing suppressed lipid accumulation in HCC cells, and that it altered the expression of MCAD and LCAD more significantly, we next focus on the regulatory effect of YY1 on MCAD and LCAD. By using both shYY1 vectors, we further confirm that YY1 silencing could significantly enhance MCAD and LCAD mRNA as well as protein levels under hypoxic condition (**Figure 1G-H**). Accordingly,* YY1* overexpression reduced the expression levels of MCAD and LCAD (**Figure S2B, Figure 1I**), confirming that YY1 negatively regulated MCAD and LCAD expression at the transcriptional level. Together, these results indicated that YY1 is a novel regulator of MCAD and LCAD expression.

### YY1 suppresses fatty acid β-oxidation by suppressing MCAD and LCAD levels

**Figure 2:**
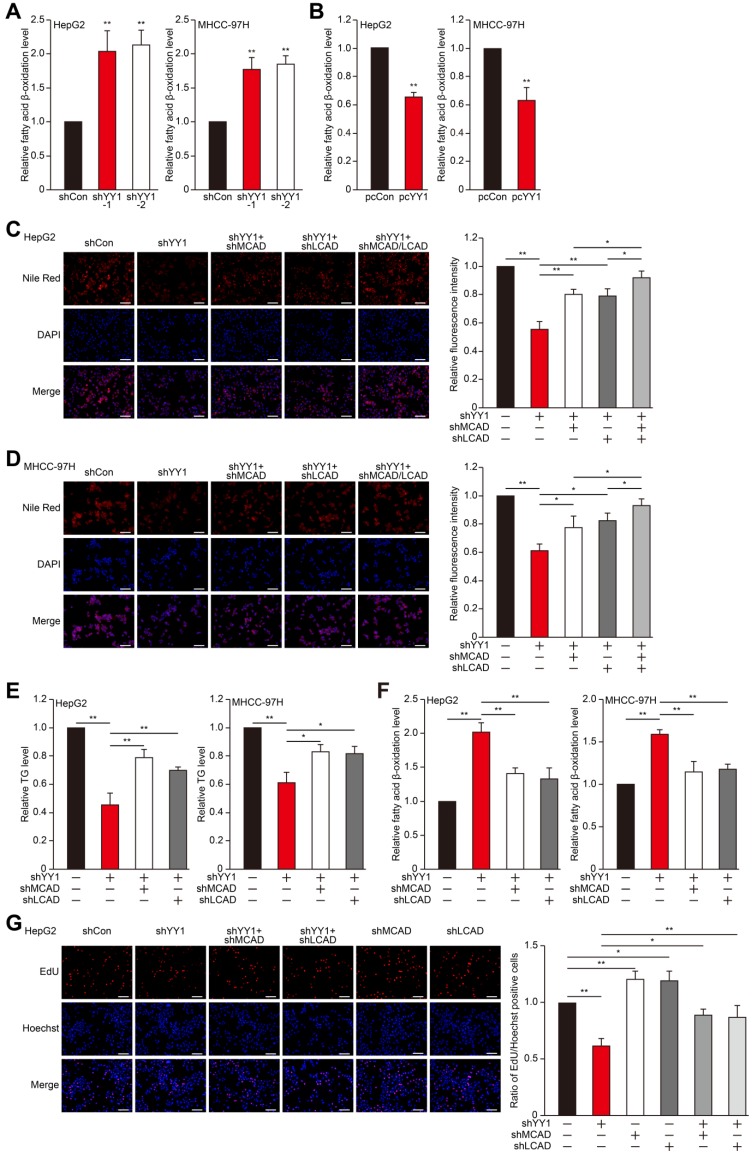
** YY1 upregulates fatty acids β-oxidation by negatively regulates the expression of MCAD and LCAD at their transcriptional levels. A-B.** The level of fatty acid β-oxidation in *YY1*-silenced (A) and *YY1*-overexpressed (B) HepG2 and MHCC-97H cells (n = 3). **C-D.** The accumulation of lipid droplets in *YY1*/*MCAD-* or *YY1*/*LCAD-*double silenced HepG2 (C) and MHCC-97H (D) cells, as examined using Nile Red staining. Representative images (left) and relative fluorescence intensity (right, n = 9) are shown. **E-F.** The levels of cellular TG (E) and fatty acid β-oxidation (F) in *YY1*/*MCAD-* or *YY1*/*LCAD-*double silenced HepG2 (left) and MHCC-97H (right) cells (n = 3). **G.** Number of proliferative *YY1*/*MCAD-* or *YY1*/*LCAD-*double silenced HepG2 cells, as determined by EdU incorporation assay. Representative images (left) and ratio of the EdU positive cells to the total cell number (right) are shown (n = 9). All experiments were performed under hypoxic condition. Cells transfected with shCon or pcCon were used as controls. Scale bars: 200 μm. Quantification data are shown as mean ± SEM of three independent experiments. pcCon: pcDNA3.1(+); *P < 0.05; **P < 0.01 (ANOVA).

MCAD and LCAD are key enzymes involved in fatty acid β-oxidation. They enhanced fatty acids degradation, leading to the suppression of lipid accumulation and subsequently, tumor cells proliferation 7. Indeed, *MCAD* and *LCAD* silencing increased lipid accumulation (**Figure S3A-E**). We next examined whether YY1 affects fatty acid β-oxidation. We found that *YY1* silencing significantly increased fatty acid β-oxidation (**Figure 2A**), whereas* YY1* overexpression decreased it (**Figure 2B**). These results indicated possible YY1 involvement in HCC cell lipid metabolism and promoting lipid accumulation by suppressing fatty acid β-oxidation.

To elucidate the roles of MCAD and LCAD in the YY1 regulatory pathway associated with lipid metabolism, we performed *YY1/MCAD*-double silencing as well as *YY1/LCAD*-double silencing (**Figure S3F**). We confirmed that *YY1/MCAD*-double silencing and *YY1/LCAD*-double silencing clearly recovered lipid accumulation suppressed by *YY1* silencing alone under hypoxic condition in HepG2 and MHCC-97H cells, while knocking down three of them recovered lipid accumulation more significantly (**Figure 2C-D**). Furthermore, *YY1/MCAD*-double silencing and *YY1/LCAD*-double silencing also restored cellular TG level (**Figure 2E**). Concomitantly, *MCAD* and *LCAD* silencing together with *YY1* significantly attenuated the increase in fatty acid β-oxidation induced by *YY1* silencing alone (**Figure 2F**). Moreover, we found that double silencing of *YY1* and either *MCAD* or *LCAD* resulted in pronounced recovery of total cell number (**Figure S3G**) and the number of proliferative cells (**Figure 2G**). These results indicated that YY1 suppressed fatty acid β-oxidation by downregulating MCAD and LCAD levels, resulting in increased lipid accumulation under hypoxic condition.

### YY1 negatively regulates PGC-1β expression

**Figure 3:**
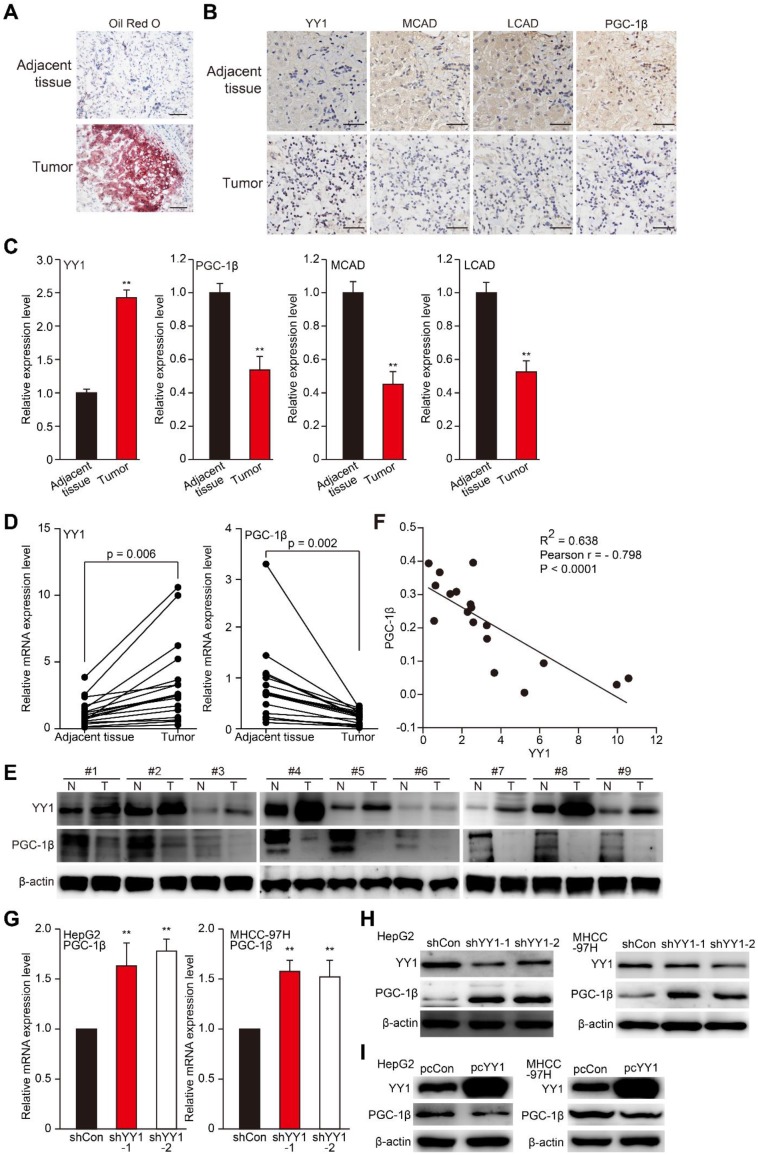
** YY1 negatively regulates *PGC-1β* at the transcriptional level in HCC cell. A.** The accumulation of lipid droplets in the clinical HCC tissue and the normal adjacent tissue, as analyzed using Oil Red O staining. Scale bars: 200 μm. **B-C.** The expression levels of YY1, MCAD, LCAD and PGC-1β in the clinical HCC tissue and the normal adjacent tissue, as analyzed by immunohistochemical staining using serial sections. Representative images (B) and the quantification results (C, n = 6) are shown. Scale bars: 40 μm. Quantification results are shown as relative to adjacent tissue. **D-E.** The mRNA (D, n = 18) and protein (E, n = 9) expression levels of YY1 and PGC-1β in clinical human HCC and the corresponding normal adjacent tissues. **F.** Correlation analysis between the mRNA expression levels of YY1 and PGC-1β in clinical HCC tissue. **G-H.** PGC-1β mRNA (G) and protein (H) expression levels in *YY1*-silenced HepG2 and MHCC-97H cells cultured under hypoxic condition, as determined using qRT-PCR (n = 3) and western blotting, respectively. **I.** PGC-1β protein expression levels in *YY1*-overexpressed HepG2 (left) and MHCC-97H (right) cells cultured under hypoxia, as determined using western blotting. Cells transfected with shCon or pcCon were used as controls. β-actin was used for qRT-PCR normalization and as western blotting loading control. Quantification data are shown as mean ± SEM of three independent experiments. pcCon: pcDNA3.1(+); **P < 0.01 (ANOVA).

To investigate the relations between YY1 and lipid accumulation in HCC, we first examined lipid accumulation, as well as levels of YY1, MCAD, LCAD, and PGC-1β in clinical HCC tissues. Previous studies demonstrated that PGC-1β is an upstream, common regulator of MCAD and LCAD that enhances their expression, and thus is crucial for fatty acid β-oxidation 7, 30. As shown in **Figure 3A-C**, compared with normal adjacent tissue, Oil Red O (**A**) and immunohistochemical staining results of clinical HCC tissues showed elevated lipid accumulation along with increased YY1 level in HCC lesions; whereas MCAD and LCAD levels were lower (**B-C**). Additionally, we found a decrease of PGC-1β level in clinical HCC tissues relative to those in normal adjacent tissues. Moreover, qRT-PCR and western blot analysis further confirmed that compared to the corresponding normal adjacent tissues, YY1 expression was upregulated in clinical HCC tissues, while PGC-1β was suppressed (**Figure 3D-E**). Furthermore, correlation analysis of YY1 and PGC-1β mRNA expression in clinical HCC tissues and normal adjacent tissues showed a negative correlation (**Figure 3F, Figure S4A**). To confirm YY1-specific regulation of *PGC-1β* expression, we examined PGC-1β expression in *YY1-*silenced HCC cells, finding that *YY1* silencing significantly upregulated PGC-1β mRNA (**Figure 3G**). In line with this, the expression level of PGC-1β protein was upregulated in *YY1-*silenced cells (**Figure 3H)**, and suppressed in *YY1-*overexpressed cells (**Figure 3I**). These results clearly suggested that YY1 negatively regulates PGC-1β at the transcriptional level, and that YY1/PGC-1β pathway might be involved in HCC progression.

### YY1 suppresses fatty acid β-oxidation by inhibiting the PGC-1β/MCAD and PGC-1β/LCAD axis

**Figure 4:**
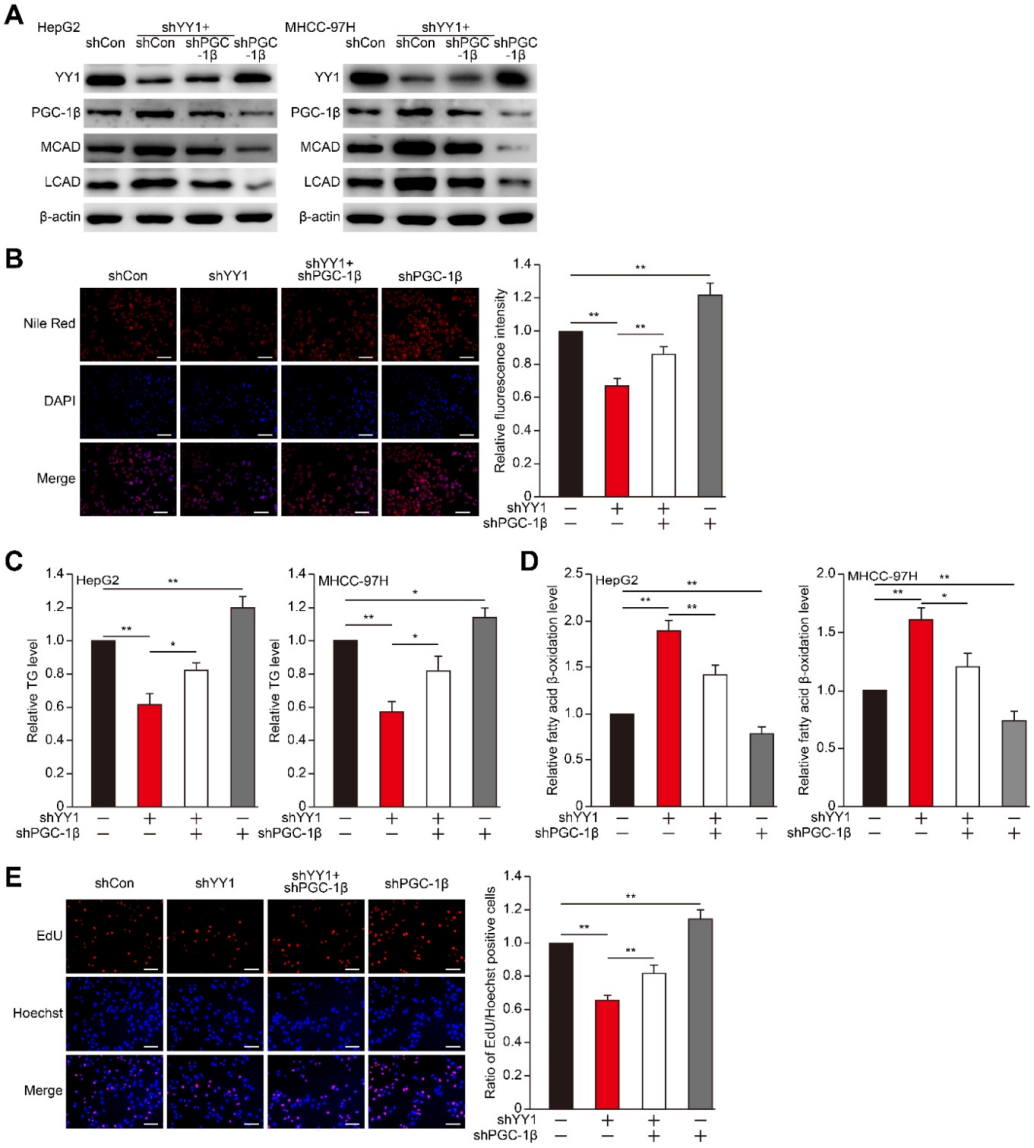
** PGC-1β is critical for YY1-induced lipid accumulation in HCC cell. A.** The protein expression levels of PGC-1β, MCAD and LCAD in *YY1*/*PGC-1β*-double silenced HepG2 (left) and MHCC-97H (right) cells, as examined using western blotting. **B.** The accumulation of lipid droplets in *YY1*/*PGC-1β*-double silenced HepG2 cells, as analyzed using Nile Red staining. Representative images (left) and relative fluorescence intensity (right, n = 9) are shown. **C-D.** The levels of cellular TG (C) and fatty acid β-oxidation (D) in *YY1*/*PGC-1β-*double silenced HepG2 (left) and MHCC-97H (right) cells (n = 3). **E.** Number of proliferative *YY1*/*PGC-1β-*double silenced HepG2 cells, as determined by EdU incorporation assay. Representative images (left) and ratio of the EdU positive cells to the total cell number (right) are shown (n = 9). All experiments were performed under hypoxic condition. Cells transfected with shCon were used as controls. β-actin was used as western blotting loading control. Quantification data are shown as mean ± SEM of three independent experiments. Scale bars: 200 μm. *P < 0.05; **P < 0.01 (ANOVA).

To examine the role of PGC-1β in YY1-regulated HCC cell lipid accumulation, we first constructed shRNA against *PGC-1β* (**Figure S4B-C**), and performed *YY1/PGC-1β* double silencing (**Figure S4D-E**). *YY1/PGC-1β* double silencing suppressed MCAD and LCAD mRNA (**Figure S4F-G**) as well as protein (**Figure 4A**) expression in both HepG2 and MHCC-97H cells. Concomitantly, *YY1*/*PGC-1β* double silencing re-induced lipid and TG accumulation suppressed by *YY1* silencing alone (**Figure 4B-C**). Furthermore, *PGC-1β* silencing cancelled the observed increase in fatty acid β-oxidation level induced by *YY1* silencing (**Figure 4D**), suggesting that YY1 negatively regulates the PGC-1β/MCAD and PGC-1β/LCAD axis to block fatty acid β-oxidation and subsequently increase lipid accumulation. As YY1 is a positive regulator of tumor cell proliferation, and that lipid accumulation is crucial for tumor cell proliferation, we further investigated the role of YY1/PGC-1β axis in HCC cell proliferation. Our results showed that *PGC-1β* silencing restored the proliferative potential (**Figure 4E**) and total cell number (**Figure S4H**) suppressed by *YY1* silencing alone. These results indicated that YY1 regulation of lipid accumulation and subsequent effect on HCC cell proliferation and tumorigenesis potential occurred through inhibition of PGC-1β expression.

### YY1 regulates PGC-1β irrespective of HIF-1α status

**Figure 5:**
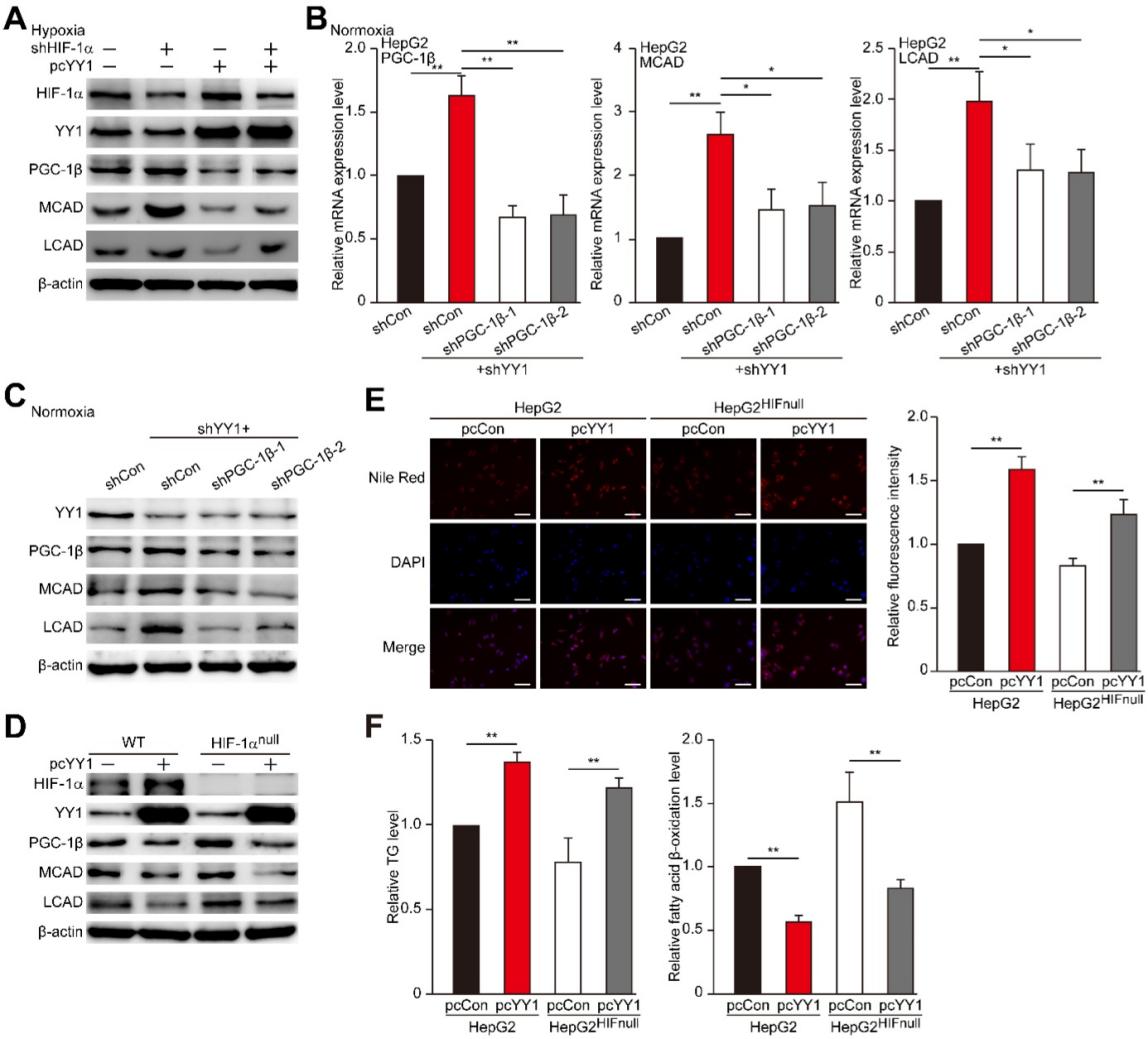
** YY1 regulates HCC cell lipid accumulation in a HIF-1α independent pathway. A.** Protein expression levels of PGC-1β, MCAD and LCAD in *HIF-1α-*silenced HepG2 cells overexpressing *YY1* cultured under hypoxic condition, as examined using western blotting. **B-C.** mRNA (B) and protein (C) expression levels of PGC1-β, MCAD and LCAD in *YY1*/*PGC-1β-*double silenced HepG2 cells cultured under normoxic condition, as determined using qRT-PCR (n = 3) and western blotting, respectively. **D.** Protein expression level of PGC-1β, MCAD and LCAD in *YY1*-overexpressed *HIF-1α*-knocked out HepG2 cells (HepG2^HIFnull^) cultured under hypoxic condition, as determined using western blotting. **E.** The accumulation of lipid droplets in *YY1*-overexpressed HepG2^HIFnull^ cells cultured under hypoxic condition, as analyzed using Nile Red staining. Representative images (left) and relative fluorescence intensity (right, n = 9) are shown. **F.** The level of cellular TG (left) and fatty acid β-oxidation (right) in *YY1*-overexpressed HepG2^HIFnull^ cells cultured under hypoxic condition (n = 3). Cells transfected with shCon or pcCon were used as controls. β-actin was used for qRT-PCR normalization and as western blotting loading control. Quantification data are shown as mean ± SEM of three independent experiments. Scale bars: 200 μm. pcCon: pcDNA3.1(+); *P < 0.05; **P < 0.01 (ANOVA).

Previous reports showed that PGC-1β is regulated by HIF-1α 7, and that YY1 stabilizes HIF-1α protein under hypoxic condition 19. Therefore, we next assessed whether YY1 regulation of PGC-1β occurs in a HIF-1α-dependent manner. Indeed, we found that overexpression of HIF-1α partially suppressed the protein expression levels of PGC-1β, MCAD and LCAD upregulated by *YY1*-silencing (**Figure S5A-B**), suggesting the presence of HIF-1α-dependent pathway in YY1 regulation on PGC-1β. To determine whether HIF-1α is compulsory in YY1/PGC-1β pathway, we examined the effect of *YY1* overexpression in* HIF-1α*-silenced cells (**Figure S5C**). As shown in **Figure 5A**, consistent with our previous report 19, *YY1* overexpression enhanced the accumulation of HIF-1α protein; however, intriguingly, our results clearly showed that similar to that in the cells with HIF-1α, *YY1* overexpression continued to significantly suppress PGC-1β level, as well as its downstream targets, even in *HIF-1α*-silenced cells. These results showed that YY1 regulation on PGC-1β could also occur in a HIF-1α-independent manner.

HIF-1α is a master regulator of the response to hypoxia, as it is stabilized under hypoxic condition while being hydroxylated at Pro402 and Pro564 under normoxic condition, leading to its ubiquitination and proteasomal degradation rapidly 36-39. Given that YY1 could also regulate PGC-1β in a HIF-1α-independent manner, we next examined the effect of *YY1* silencing on *PGC-1β* under normoxic condition, in which HIF-1α was degraded and almost undetectable (**Figure S5D**). Concomitant with the effect on *HIF-1α*-silenced cells, we observed increased levels of PGC-1β, MCAD, and LCAD in *YY1*-silenced cells under normoxic condition, whereas silencing both *YY1* and *PGC-1β* attenuated MCAD and LCAD levels (**Figure 5B-C**). Similarly, we found that *PGC-1β* silencing restored TG and fatty acid β-oxidation level altered by *YY1* silencing under normoxic condition (**Figure S5E**). These results clearly indicated that YY1 could regulate HCC cell lipid accumulation through PGC-1β/MCAD and PGC-1β/LCAD axis even when HIF-1α was absence, suggesting the presence of HIF-1α-independent pathway in YY1 regulation of PGC-1β-induced fatty acid β-oxidation.

To further confirm this finding, we constructed a HIF-1α-null HepG2 cell line (HepG2^HIFnull^) using the CRISPR/Cas9 method (**Figure S5F**). We then overexpressed* YY1* in HepG2^HIFnull^ cells, and analyzed its effect on the levels of PGC-1β and its downstream targets under hypoxic condition. We found that similar to results in wild-type HepG2 cells, *YY1* overexpression in HepG2^HIFnull^ cells significantly suppressed PGC-1β, MCAD, and LCAD expression in their mRNA (**Figure S5G**) and protein (**Figure 5D**) levels. Furthermore, YY1 overexpression significantly upregulated lipid accumulation, as well as TG level, in HepG2^HIFnull^ cells under hypoxic condition, most likely due to the inhibition of fatty acid β-oxidation (**Figure 5E-F**). These results clearly demonstrated the presence of a HIF-1α-independent pathway involved in YY1-dependent fatty acid β-oxidation inhibition.

### YY1 binds to the PGC-1β promoter and suppresses its transcription

**Figure 6:**
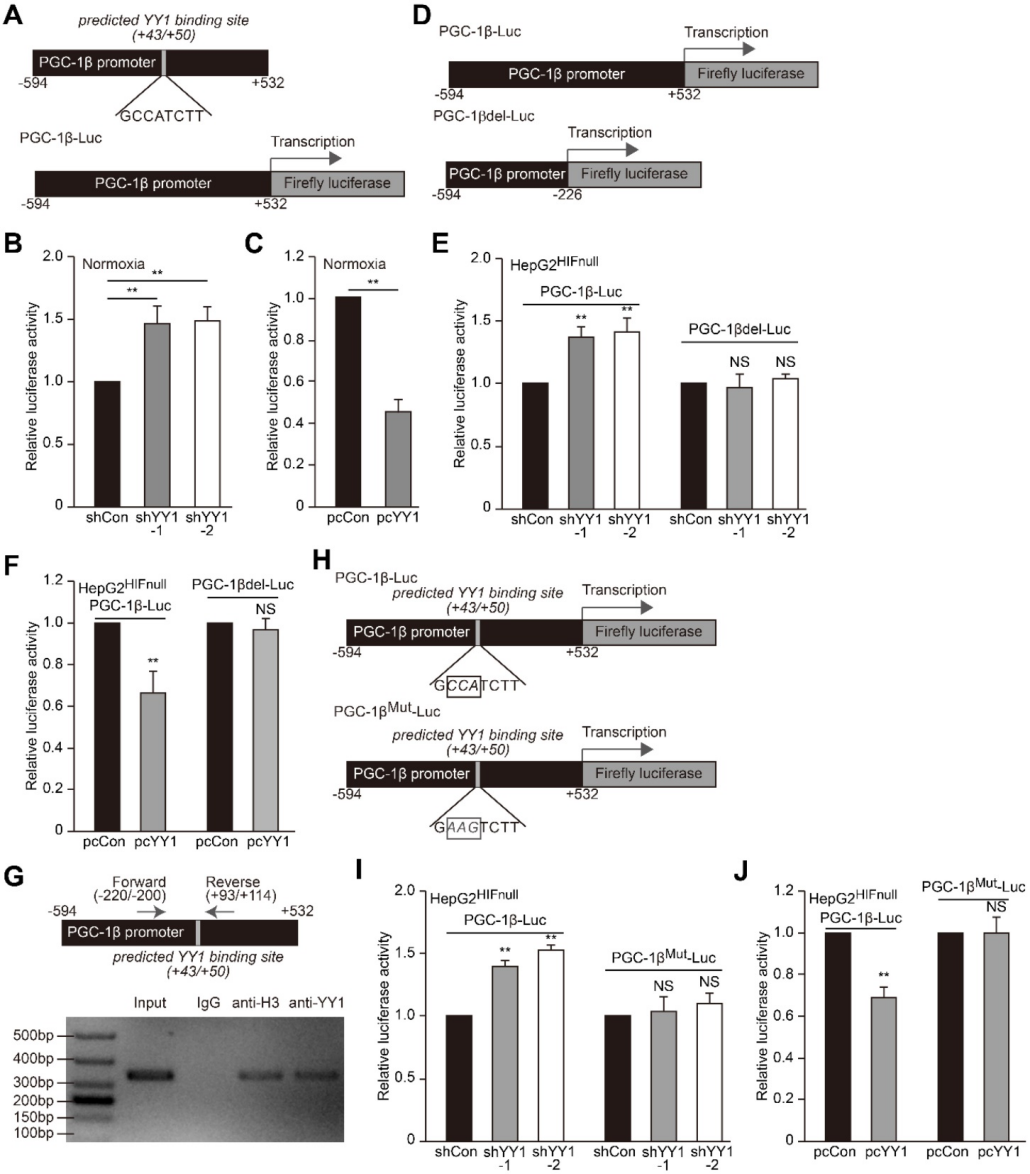
** YY1 binds to PGC-1β promoter and enhances its transcription independently of HIF-1α. A.** Schematic diagrams of the predicted YY1 binding site in *PGC-1β* promoter and the luciferase reporter bringing *PGC-1β* promoter (PGC-1β-Luc). **B-C.** The activity of PGC-1β-Luc in *YY1*-silenced (B) and *YY1-*overexpressed (C) HepG2 cells cultured under normoxic condition, as analyzed by using dual luciferase assay (n = 3). **D.** Schematic diagram of firefly luciferase reporter bringing the *PGC-1β* promoter lacking YY1 binding site (PGC-1βdel-Luc). **E-F.** The activities of PGC-1β-Luc and PGC-1βdel-Luc in *YY1*-silenced (E) and *YY1*-overexpressed (F) HepG2^HIFnull^ cells cultured under hypoxic condition, as analyzed using dual luciferase assay (n = 3). **G.** Binding of YY1 to the promoter region of *PGC-1β* in HepG2 cells as examined using chromatin immunoprecipitation assay with anti-YY1 antibody followed by PCR. Location of the primer set (top) and the length of the amplicon (bottom) are shown. **H.** Schematic diagram of the firefly luciferase reporter bringing *PGC-1β* promoter with mutated predicted YY1 binding site (PGC-1β^Mut^-Luc). Wild-type nucleotides are shown in black, and mutated ones are shown in gray. **I-J.** The activities of PGC-1β-Luc and PGC-1β^Mut^-Luc in *YY1*-silenced (I) and *YY1*-overexpressed (J) HepG2^HIFnull^ cells cultured under hypoxic condition, as analyzed using dual luciferase assay (n = 3). Cells transfected with shCon or pcCon were used as controls. Quantification data are shown as mean ± SEM of three independent experiments. pcCon: pcDNA3.1(+); **P < 0.01; NS: not significant (ANOVA).

As YY1 negatively regulates PGC-1β level in the absence of HIF-1α, we next investigated whether YY1 directly regulates *PGC-1β* transcription. According to the UCSC genome browser, we identified a YY1 consensus binding site in the *PGC-1β* promoter region (+43 to +50, **Figure 6A upper panel**) 40; thus we first examined the effect of manipulating *YY1* expression on *PGC-1β* transcription using a luciferase reporter bringing the -594 to +532 region of the *PGC-1β* promoter (PGC-1β-Luc, **Figure 6A lower panel**). A dual luciferase assay using the reporter vector revealed that under normoxic condition, *YY1* silencing significantly induced PGC-1β-Luc activity, whereas* YY1* overexpression significantly reduced it (**Figure 6B-C**), indicating that YY1 might negatively regulate *PGC-1β* transcription. Next we constructed a luciferase-reporter vector without YY1 binding site harboring the -594 to -226 region of the *PGC-1β* promoter (PGC-1βdel-Luc;** Figure 6D**), followed by evaluation of the effect of *YY1* silencing. The results showed that while *YY1* silencing resulted in the increase of PGC-1β-Luc activity in HepG2^HIFnull^ cells under hypoxic condition, it failed to increase PGC-1βdel-Luc activity (**Figure 6E**). Consistently, *YY1* overexpression significantly suppressed the activity of PGC-1β-Luc but not PGC-1βdel-Luc activity (**Figure 6F**). These results suggested that YY1 targeted the *PGC-1β* promoter in a HIF-1α-independent manner, and that the -227 to +532 region of the *PGC-1β* promoter was essential for regulation of YY1 on *PGC-1β*.

We then analyzed whether YY1 binds the predicted site within the *PGC-1β* promoter. We first performed a chromatin immunoprecipitation (ChIP) assay using the indicated primers flanking the predicted YY1 binding site (**Figure 6G**, **upper panel**), finding that the corresponding promoter region was detected in the chromatin immunoprecipitated using the anti-YY1 antibody (**Figure 6G**, **lower panel**). This result indicated that YY1 binds to the -220 to +114 region of the *PGC-1β* promoter. To assess function of the predicted YY1-binding site, we constructed a mutant PGC-1β-Luc reporter (PGC-1β^Mut^-Luc) by mutating 3 nucleotides in the predicted YY1-binding site (G*CCA*TCTT to G*AAG*TCTT) (**Figure 6H**). As shown in **Figure 6I**, while *YY1-*silencing robustly induced the activity of the wild-type PGC-1β-Luc reporter in HepG2^HIFnull^ cells cultured under hypoxic condition, this effect was diminished upon use of the PGC-1β^Mut^-Luc reporter. In agreement with this result, compared to cells transfected with control vector, *YY1* overexpression failed to suppress the activity of the PGC-1β^Mut^-Luc reporter while significantly reduce that of the PGC-1β-Luc reporter (**Figure 6J**). These results suggested that YY1 directly regulates *PGC-1β* transcription through binding its promoter in the +43 to +50 region; and that this regulation occurs independent of HIF-1α status.

### The YY1/PGC-1β axis is critical for hepatocarcinogenesis potential

**Figure 7:**
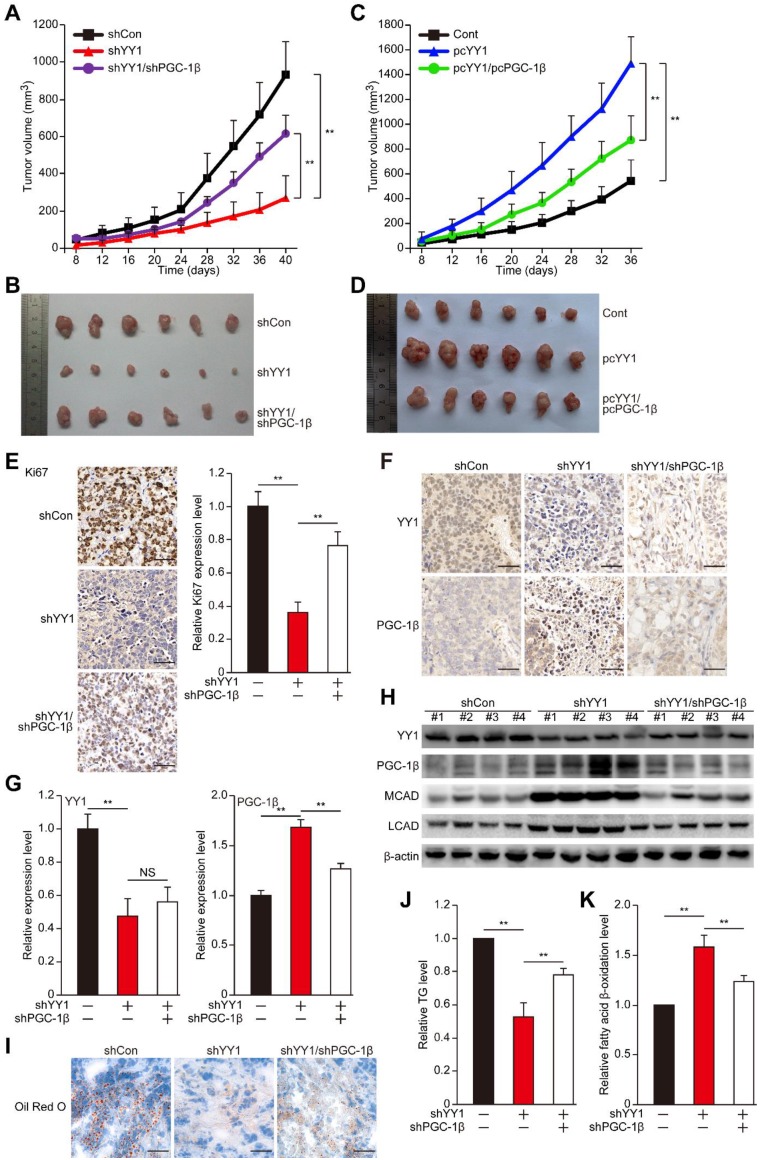
** YY1 mediates hepatocarcinogenesis potential by negatively regulates PGC-1β. A-B.** Hepatocarcinogenesis potential of control (shCon), *YY1*-silenced (shYY1) and *YY1/PGC-1β*-double silenced (shYY1/shPGC-1β) MHCC-97H stable cell lines were examined *in vivo* by subcutaneous injection into Balb/c-nu/nu mice (n = 6). Volume of the tumors formed at indicated time points (A) and the appearance (B) are shown. **C-D.** Hepatocarcinogenesis potential of control (Cont), *YY1*-overexpressed (pcYY1) and *YY1/PGC-1β*-double overexpressed (pcYY1/pcPGC-1β) MHCC-97H stable cell lines were examined *in vivo* by subcutaneous injection into Balb/c-nu/nu mice (n = 6). Volume of the tumors formed at indicated time points (C) and the appearance (D) are shown. **E.** Proliferative cells in the tissue section of xenografted tumors in Balb/c-nu/nu mice injected with the indicated cell lines, as stained using Ki67. Scale bars: 40 μm. Representative images (left) and quantification results (right, n = 6) are shown. **F-G.** Immunohistochemical staining images against YY1 (top) and PGC-1β (bottom) in the tissue section of xenografted tumors in Balb/c-nu/nu mice injected with the indicated cell lines. Scale bars: 40 μm. Representative images (F) and relative expression level (G) are shown (n = 6). Quantification was performed by counting the ratio of the positive cells to total cell number, and the results are shown as relative to control. **H.** YY1, PGC1-β, MCAD and LCAD protein expression levels in the xenografted tumors in Balb/c-nu/nu mice injected with the indicated cell lines were examined using western blotting. **I.** The accumulation of lipid droplets in the tissue section of xenografted tumors in Balb/c-nu/nu mice injected with the indicated cell lines were stained using Oil Red O staining. **J-K.** The level of cellular TG (J) and fatty acid β-oxidation (K) in the xenografted tumors in Balb/c-nu/nu mice injected with the indicated cell lines. Cells transfected with shCon or pcEF9-puro were used as controls. β-actin was used as western blotting loading control. Quantification data are shown as mean ± SEM of three independent experiments. **P < 0.01; NS: not significant (ANOVA).

Next we examined the role of YY1/PGC-1β axis in hepatocarcinogenesis. While knocking down *YY1* suppressed HCC cells colony formation potential, double knocked down of YY1 and *PGC-1β* restored it (**Figure S6**), indicating that PGC-1β might be involved in *YY1*-mediated hepatocarcinogenesis. To confirm this, finally we examined the pathological function of the YY1/PGC-1β pathway in hepatocarcinogenesis potential *in vivo*. To this end, we constructed stable *YY1-*silenced and *YY1*/*PGC-1β*-double silenced cell lines (**Figure S7A**), as well as *YY1-*overexpression and *YY1*/*PGC-1β*-double overexpressed cell lines (**Figure S7B**) using MHCC-97H cells. Xenograft experiments showed that *YY1* silencing reduced the tumorigenesis potential of MHCC-97H cells; however, silencing of both *YY1* and *PGC-1β* significantly restored this potential (**Figure 7A-B**). Furthermore, while *YY1-*overexpression robustly enhanced tumorigenesis potential, overexpression of both *YY1* and *PGC-1β* reduced it (**Figure 7C-D**), suggesting that YY1/PGC-1β axis is crucial for promoting hepatocarcinogenesis. Moreover, Ki67 staining results also showed that the number of proliferative cells was conspicuously lower in xenografted tumors originating from *YY1-*silenced MHCC-97H cells, while *PGC-1β*-silencing significantly restored it (**Figure 7E**). Consistent with this finding, immunohistochemical staining results also revealed an upregulation of PGC-1β level in xenografts generated from *YY1-*silenced cells, whereas silencing of both *YY1* and *PGC-1β* cancelled this effect (**Figure 7F-G**). Western blotting analysis also confirmed these results, as inhibition of YY1 expression correlating with significant increase in PGC-1β, MCAD and LCAD protein levels (**Figure 7H**).

To further reveal the relation between YY1/PGC-1β-induced tumorigenesis and lipid accumulation *in vivo*, we next examined the lipid accumulation, TG and fatty acid β-oxidation in xenografted tumors. Significant decreases of lipid accumulation and TG were observed in the xenografted tumors originating from *YY1-*silenced cells; however, the amounts of lipid accumulation and TG were restored in tumors originating from *YY1*/*PGC-1β*-double silenced cells (**Figure 7I-J**). In line with this, *YY1/PGC-1β*-double silencing significantly attenuated the increase of fatty acid β-oxidation in xenografted tumors induced by *YY1* silencing alone (**Figure 7K**). These results clearly showed a negative correlation between YY1 and PGC-1β expression, as well as the importance of YY1/PGC-1β axis in lipid metabolism and tumor progression of hepatocellular carcinoma.

**Figure 8:**
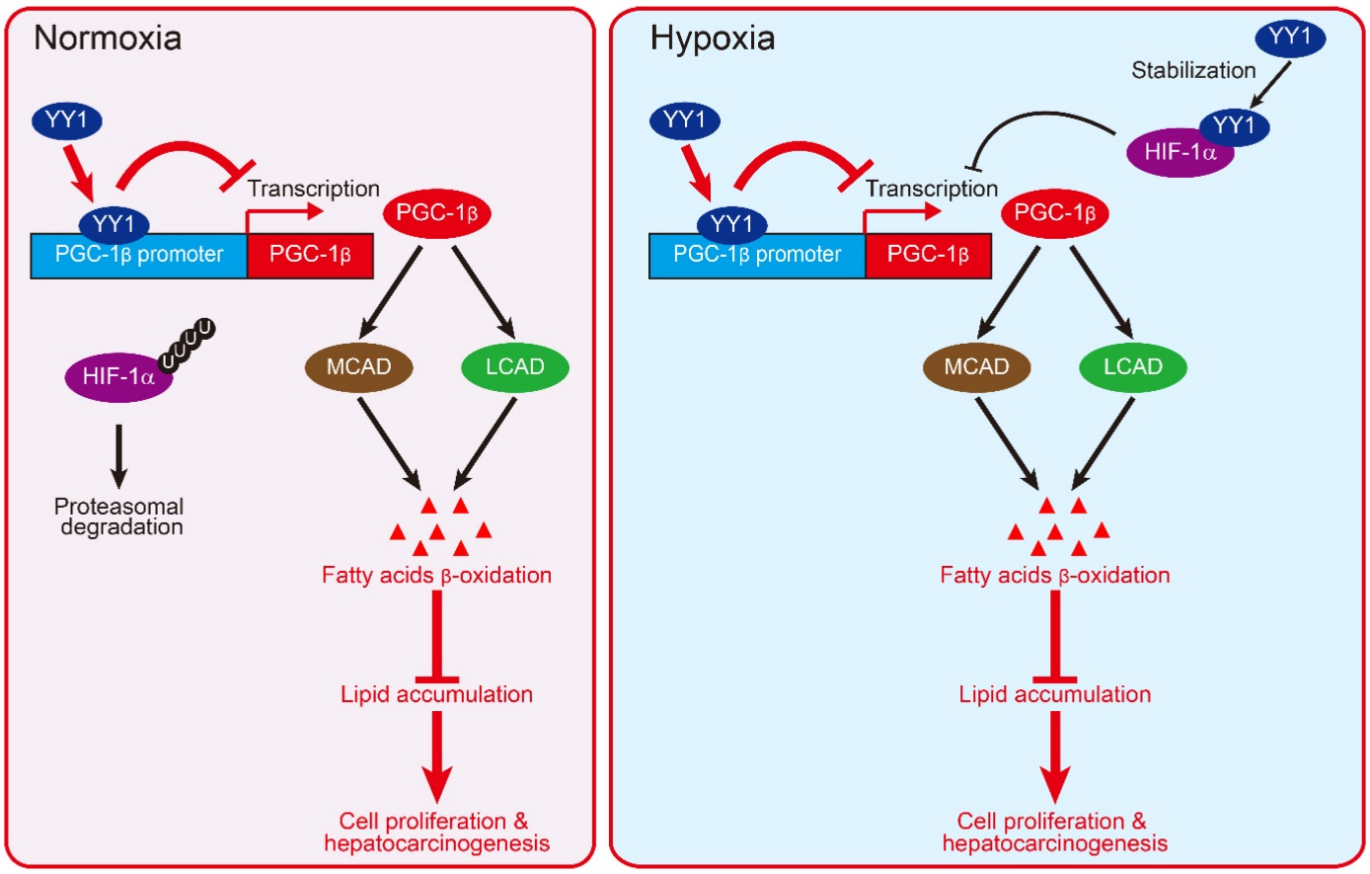
** Schematic diagram showing the mechanism of YY1/PGC-1β axis-mediated HCC cells lipid metabolism in both normoxia and hypoxia**.

Together, our results described a novel regulatory mechanism involved in HCC cell lipid metabolic reprogramming. We found that YY1 suppressed *PGC-1β* transcription by directly binds to its promoter, resulting in subsequent suppression of MCAD and LCAD levels and decreased fatty acid β-oxidation irrespective of HIF-1α status. This in turn elevated lipid accumulation, which is a crucial driver of hepatocarcinogenesis (**Figure 8**).

## Discussion

Metabolic reprogramming is a characteristic of tumor cells and is essential for supporting their rapid proliferation, as it provides tumor cells with several benefits, including macromolecule biosynthesis, adaptation to the microenvironment, and an ability to cope with oxidative stress 1, 2, 41. Lipid metabolic reprogramming enables tumor cells to meet the demand of their highly proliferative growth 42, and is an important driving force for HCC development 7, 35. Specifically, lipid metabolic reprogramming in tumor cells induces aberrant lipid accumulation 3. Consequently, this facilitates tumorigenesis by providing tumor cells with both the energy and materials necessary for the biosynthesis of signaling molecule such as arachidonic acid, as well as phospholipids as the major component of cell membranes 4, 43. In the present study, our findings reveal that the transcription factor YY1 is critical for lipid metabolism in HCC cells, as it negatively regulates fatty acid β-oxidation by directly binds to the *PGC-1β* promoter and suppresses its transcription under both normoxic and hypoxic conditions. YY1-mediated downregulation of PGC-1β expression in turn attenuates MCAD and LCAD levels, leading to the suppression of fatty acid β-oxidation and subsequently lipid accumulation. Furthermore, as previous studies revealed that PGC-1β is crucial for muscle-cell metabolism and suppressing inflammation 44, 45, our results also suggest the possibility of YY1 involvement in other biological processes apart from tumorigenesis through the YY1/PGC-1β axis.

Previous studies showed that aberrant YY1 expression is observed in cancers, including colon, breast, liver and pancreatic cancers 23-27. YY1 has been implicated in tumor progression, as it could enhance cell proliferation by facilitating interactions between the tumor suppressor p53 and its upstream negative regulator mouse double-minute homolog-2, thereby inducing p53 degradation 18. Moreover, YY1 destabilizes p53 and blocks p53-mediated transcriptional activity by disrupting its interaction with p300 17. Furthermore, YY1 supports tumor cell proliferation and survival by inducing tumor angiogenesis 16, 19. Despite its importance as an oncogene, the role of YY1 in tumor cell lipid metabolism remains unraveled. Our present study elucidates a novel, critical role of YY1 in regulating tumor cell lipid homeostasis, especially by regulating fatty acid β-oxidation, linking up YY1 with tumor cell lipid metabolic reprogramming, another characteristic of tumor cell which is critical for supporting its survival, tumor progression, and metastasis 7, 46, 47. YY1 is a transcription factor crucial for the regulation of various genes 48, and furthermore, a recent report revealed that it could regulate gene expression as a structural regulators of enhancer-promoter loops 20. Hence, YY1 might also regulate lipid metabolism-related factors at their transcriptional level, indicating that although further investigation is needed, there might be alternative pathways of YY1 regulation on tumor cells lipid metabolism. Nevertheless, our findings showed for the first time the crucial role of YY1/PGC-1β pathway in tumor cells lipid metabolism.

HIF-1α is a factor induced by hypoxia. It regulates the transcription of various genes involved in cell proliferation and survival, metabolic reprogramming, and other functions that support cell-specific adaptation to the tumor hypoxic microenvironment. Huang *et al* reported that HIF-1α suppresses FAO and enhances lipid accumulation 7. On the other hand, our previous study has shown that under hypoxic condition, YY1 inhibits HIF-1α proteasomal degradation, thereby stabilizing HIF-1α protein level 19. In the present study, we show that YY1 suppresses *PGC-1β* transcription even in the absence of HIF-1α by directly binds to its promoter. This enables the YY1/PGC-1β axis to regulate lipid accumulation in HCC cells not only under hypoxic condition in both HIF-1α-dependent and -independent manners, but also under the presence of oxygen, during which HIF-1α is hydroxylated and degraded (**Figure 8**). The ability of YY1 to regulate tumor cell lipid metabolism in a HIF-1α-independent manner also provides evidence of the importance of HIF-1α-independent pathways in tumorigenesis.

Our results reveal that YY1 could induce HCC cell lipid accumulation under both hypoxic and normoxic conditions. Due to the rapid proliferation of tumor cells, oxygen pressure in tumor tissues is negatively correlated with their distance from blood vessels, thereby resulting in their inevitable exposure to hypoxic condition. Tumor cells not only adapt to hypoxic microenvironments, but hypoxia in turn supports tumor development. However, not all cells in tumor tissue are consistently exposed to hypoxic condition. A previous study reported that in tumor tissue with a diameter < 1 mm, oxygen could be supplied by diffusion from blood vessels 49. Furthermore, tumor cells secrete various angiogenic factors that induce the formation of new blood vessels in order to increase their supply of oxygen and nutrients; although, the abnormal architecture and patterns of tumor vasculature results in unstable oxygen supply 50. In the present study, our results reveal that the YY1/PGC-1β axis regulates HCC cell lipid metabolic reprogramming under both normoxic and hypoxic conditions, suggesting that this pathway is common in tumor cells, regardless of their location in tumor tissue. Additionally, this regulatory pathway likely ensures continuous lipid accumulation without being affected by a fluctuating oxygen supply. This pathway might also enable enhanced lipid accumulation in both early-stage tumor with relatively smaller size, and in late-stage tumor with relatively larger size. Furthermore, previous studies reported both increased YY1 expression and lipid accumulation in NAFLD 29, 51, which can progress to cirrhosis and subsequently HCC 10, 52. Therefore, although further investigation is required, our results demonstrate the possibility that activation of the YY1/PGC-1β axis might be crucial for liver metabolic disorder diseases, and that it might be a potential driver of HCC development from hepatic steatosis.

### Conclusions

In this study, we demonstrate a novel role for YY1 in promoting hepatocellular carcinoma progression by altering HCC cell lipid metabolism independent of oxygen pressure. These results not only provide insight regarding the molecular mechanism of tumor cell lipid metabolism, but also a new perspective regarding the function of YY1 in tumor progression. Therefore, our study provides evidences regarding the potential of YY1 as a target for anti-tumor therapies based on targeting tumor cell lipid metabolism.

## Supplementary Material

Supplementary figures and tables.

## Abbreviations

HCC: hepatocellular carcinoma
YY1: yin yang 1
YY2: yin yang 2
PGC-1β: peroxisome proliferator-activated receptor gamma coactivator-1β
FAS: fatty acid synthesis
FAO: fatty acid oxidation
HIF-1α: hypoxia-inducible factor-1α
NAFLD: non-alcoholic fatty liver disease
NASH: non-alcoholic steatohepatitis
TG: triglyceride
MCAD: medium-chain acyl-CoA-dehydrogenase
LCAD: long-chain acyl-CoA dehydrogenase
HMGCR: 3-hydroxy-3-methylglutaryl-CoA-reductase
ChIP: chromatin immunoprecipitation
