# Supplementary material for: Yin Yang 1 facilitates hepatocellular carcinoma cell lipid metabolism and tumor progression by inhibiting PGC-1β-induced fatty acid oxidation
Source: Theranostics. 2019 Oct 14;9(25):7599–615. doi: 10.7150/thno.34931 (PMC6831470; doi:10.7150/thno.34931)
Supplement: Supplementary file 1 — Supplementary figures and tables. [file thnov09p7599s1.pdf]

Supplementary Materials

Yin Yang 1 facilitates hepatocellular carcinoma cell lipid metabolism and tumor progression by inhibiting PGC-1β-induced fatty acid oxidation

Yanjun Li, Vivi Kasim*, Xuesong Yan, Lang Li, Ian Timothy Sembiring Meliala, Can Huang, Zhuolin Li, Ke Lei, Guanbin Song, Xiaodong Zheng, Shourong Wu*

*Email: vivikasim@cqu.edu.cn (V.K.)
shourongwu@cqu.edu.cn (S.W.)

Supplementary Materials and Methods

Triglyceride (TG) measurement

Cells were transfected with indicated shRNA expression vectors or overexpression vectors as described. For cellular TG measurement, cells were collected, re-suspended in ice-cold PBS and homogenized using sonication. For xenograft tumor TG measurement, tissue was ground with liquid nitrogen, re-suspended in ice-cold PBS and homogenized using sonication. Equal volume of lysates was used to measure TG levels using Biochemical Triglyceride Determination Kit (NJJC Bio, Nanjing, China). The values were then normalized with total protein amount determined using BCA Protein Assay Kit (Beyotime).

Nile Red staining and Oil Red O staining

For Nile Red staining, cultured cells were fixed using 4% paraformaldehyde and then stained with 0.05 mg/ml Nile Red (Sigma-Aldrich, St. Louis, MO) for 15 min followed by PBS washing and DAPI staining. Images were taken by DMI6000B (Leica, Heidelberg, Germany). Quantification was analyzed using ImageJ software. The values were shown as the fluorescence intensity normalized by the total area of the cell.

For Oil Red O staining of cultured cells, cells were fixed using 10% paraformaldehyde and then stained with 0.5% Oil Red O (Sangon Bio, Shanghai, China) for 20 min followed by 60% isopropanol washing, and hematoxylin (Beyotime) staining. For Oil Red O staining of tissue sections, frozen sections from human HCC, normal adjacent tissues and xenografted tumor tissues (8 μm thickness) were fixed in 10% formalin, washed with 60% propylene glycerol, and then stained with 0.5% Oil Red O in propylene glycerol for 10 min at 60℃.

RNA extraction and quantitative RT-PCR (qRT-PCR) analysis

Total RNA was extracted with Trizol (Invitrogen Life Technologies) according to the manufacturer’s instruction. cDNA was obtained from 1 μg RNA by reverse-transcription using the PrimeScript RT Reagent Kit with gDNA Eraser (Takara Bio). qRT-PCR was performed using SYBR Premix Ex Taq (Takara Bio) to assess mRNA expression levels. The

sequences of the primers used for qRT-PCR were shown in **Table S1**. β-actin was used to normalize sample amplifications.

Western blotting

Cultured cells were collected and lysed with RIPA lysis buffer with protease inhibitor and phosphatase inhibitor cocktail (complete cocktail; Roche Applied Science, Mannheim, Germany). For clinical specimens, the frozen specimens were homogenized with RIPA lysis buffer with protease inhibitor and phosphatase inhibitor cocktail (complete cocktail; Roche Applied Science) to obtain protein extracts. Proteins were separated using SDS PAGE, and transferred to polyvinylidene fluoride (PVDF) membrane (Millipore, Billerica, MA). The antibodies used are shown in **Table S2**. Immunoblotting with anti-β-actin antibody was conducted to ensure equal protein loading. Signals were detected using SuperSignal West Femto Maximum Sensitivity Substrate (Thermo Scientific, Waltham, MA).

Immunohistochemistry

Fresh human HCC, normal adjacent tissues and xenografted tumors were fixed using 4% paraformaldehyde for overnight prior to being embedded in paraffin and sectioned at 4 μm thickness using a cryostat. Sections were then dewaxed using xylene and rehydrated, and subjected to immunohistochemical staining. Briefly, the tissue sections were incubated with primary antibodies for 1 h, following by incubation with corresponding second antibodies conjugated with horse-radish peroxidase. Visualization was performed using a DAB Kit (DAKO, Beijing, China) under microscope. The nuclei were then counterstained with hematoxylin (Beyotime), followed by dehydration and coverslip mounting. The antibodies used were listed in **Table S2**. Images were taken using Pannoramic Midi (3DHistech, Budapest, Hungary). Quantification was performed using ImageJ software. Relative expression levels of YY1, PGC-1β, and Ki67 (nucleic proteins) were shown as the ratio of YY1, PGC-1β or Ki67 positive cells to the ratio of the hematoxylin-positive cells; while

those of MCAD and LCAD (cytoplasmic proteins) were shown as the signal intensity normalized by the total area of the cells.

Chromatin immunoprecipitation (ChIP) assay

ChIP analysis was performed using the ChIP-IT Express (Active Motif, Carlsbad, CA) according to the manufacturer's instructions. Briefly, formaldehyde (final concentration 1%) was added to the culture medium of HepG2 cells overexpressing YY1 to crosslink proteins to DNA. Cells were then collected, and the pellets were treated with Lysis Buffer prior to sonication to shear DNA into 0.2–1.0 kb fragments. After the cellular debris was removed, the chromatins were then immunoprecipitated using protein G Magnetic Beads and anti-YY1 antibody, anti-Histone H3 antibody as positive control or normal rabbit IgG as negative control. Chromatin was then de-crosslinked for 15 min at 95 ℃ prior to treatment with RNase A and proteinase K, and subjected to PCR analysis using PrimeSTAR Max (Takara Bio). The sequence of the forward primer used was 5’-CCA AGC GCT GAA AGG AAG AT-3’; while that of the reverse primer was 5’-GAA GTG TGA GGT CGA TCT GT-3’.

EdU incorporation assay and colony formation assay

Cells were transfected with indicated shRNA expression vectors and selected using puromycin as described above. EdU incorporation and staining were performed using Cell-Light™ EdU Apollo®488 In Vitro Imaging Kit (RiboBio, Guangzhou, China) according to the manufacturer's instruction. Nuclei were stained with Hoechst. Images were taken with DMI6000B (Leica). Quantification of EdU positive and Hoechst positive cells was performed using Microsystems LAS AF-TCS MP5 (Leica), and the results are shown as the ratio of EdU positive cells to Hoechst positive cells. For colony formation assay, 200 cells were cultured in a six-well plate for 12 days. Cells were then fixed with 30% paraformaldehyde and stained with crystal violet. The colonies were then counted. The investigator was blinded during the assessment.

Dual luciferase assay

Cells were co-transfected with indicated shRNA expression vectors or overexpression vectors, indicated firefly luciferase-based reporter vector, and *Renilla* luciferase expression vector pRL-SV40 as an inner control (Promega). 24 h later, luciferase reporter activities were analyzed by using Dual Luciferase Reporter Assay (Promega).

Cell counting assay

Cells transfected with indicated shRNA expression vectors and selected by using puromycin were re-seeded in a 96-well cell culture dish at a density of 3×10^3 cells/well. Cell numbers were counted at indicated time points using colorimetric assays with 3-(4,5-dimethylthiazol -2-yl)-5-(3-carboxymethoxyphenyl)-2-(4-sulfophenyl)-2H-tetrazolium (MTS, Promega) in accordance with the manufacturer's instructions.

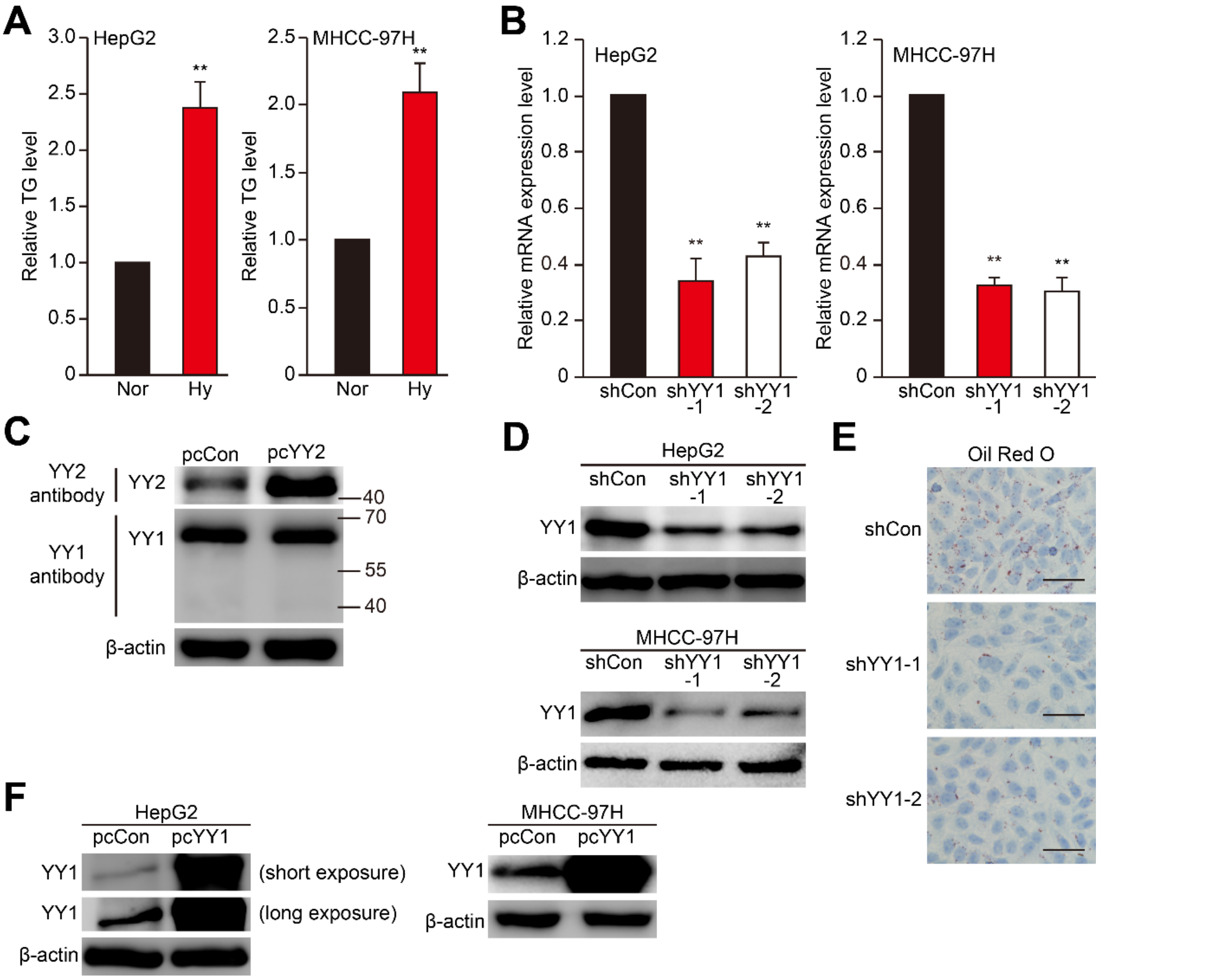

Figure S1. YY1 induces HCC cell lipid accumulation. A. Relative amount of cellular triglyceride (TG) in HepG2 (left) and MHCC-97H (right) cells cultured under normoxic (Nor) or hypoxic (Hy) condition (n = 3). **B.** YY1 mRNA expression level in HepG2 (left) and MHCC-97H (right) cells transfected with shRNAs targeting *YY1* under hypoxic condition, as determined using quantitative reverse-transcribed PCR (qRT-PCR) (n = 3). **C.** YY1 and YY2 protein expression levels in HepG2 cells overexpressing YY2, as determined using western blotting. **D.** YY1 protein expression level in HepG2 (top) and MHCC-97H (bottom) cells transfected with shRNAs targeting *YY1* under hypoxic condition, as determined using western blotting. **E.** The accumulation of lipid droplets in *YY1*-silenced HepG2 cells under hypoxic condition, as determined using Oil Red O staining. Scale bars: 200 μm. **F.** YY1 protein expression level in HepG2 (left) and MHCC-97H (right) cells transfected with *YY1* overexpression vector under hypoxic condition, as determined using western blotting. Cells transfected with control vector (shCon or pcCon) were used as controls. β-actin was used for qRT-PCR normalization and as western blotting loading control. Quantification data are shown as mean ± SEM of three independent experiments. pcCon: pcDNA3.1(+); $^{**}P < 0.01$ (ANOVA).

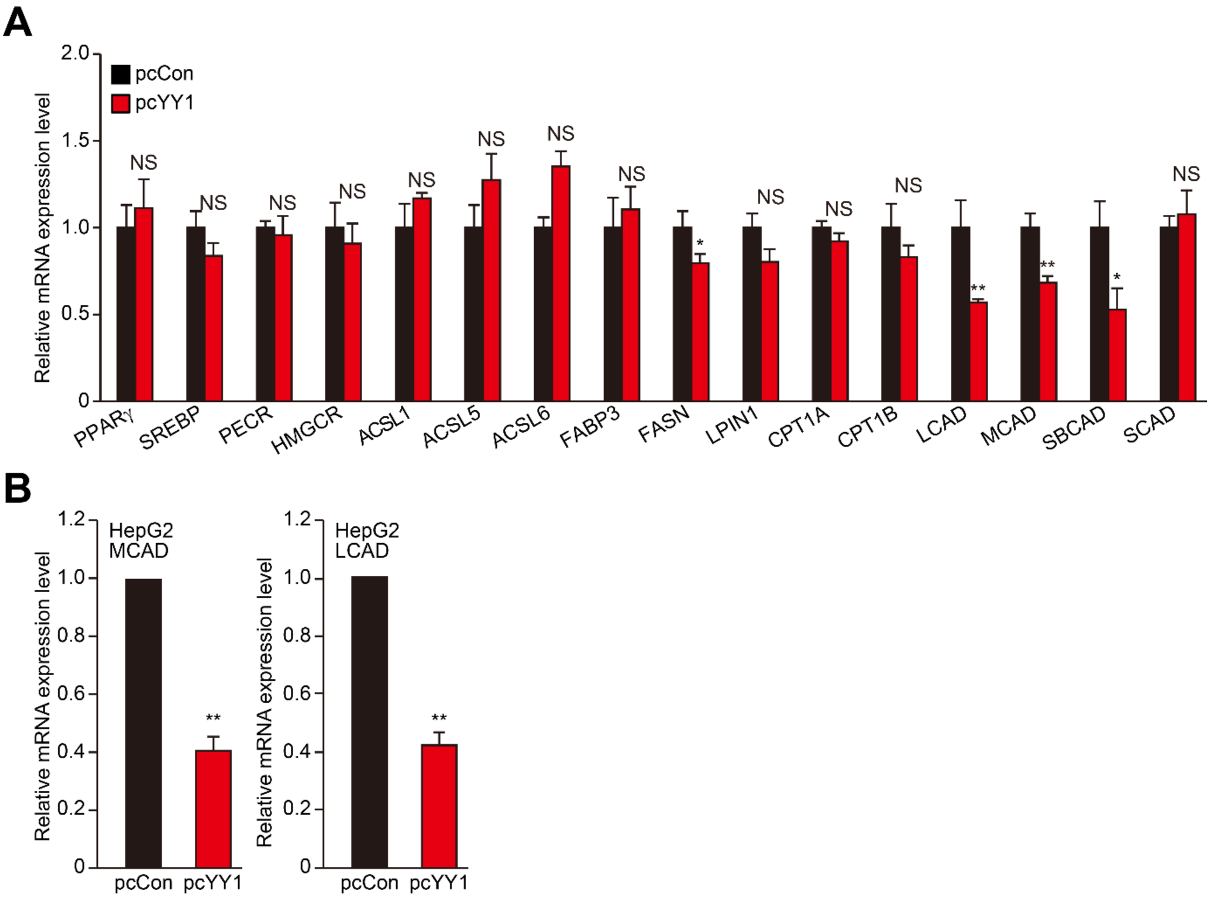

Figure S2. YY1 suppresses the expression levels of MCAD and LCAD. A. mRNA expression levels of various lipid metabolic-associated factors in *YY1*-overexpressed HepG2 cells. Representative data are shown (n = 3). **B.** MCAD and LCAD mRNA expression levels in *YY1*-overexpressed HepG2 cells, as determined using qRT-PCR (n = 3). All experiments were performed under hypoxic condition. Cells transfected with control vector (pcCon) were used as controls. β-actin was used for qRT-PCR normalization. Quantification data are shown as mean ± SEM of three independent experiments. pcCon: pcDNA3.1(+); *$P < 0.05$; **$P < 0.01$; NS: not significant (ANOVA).

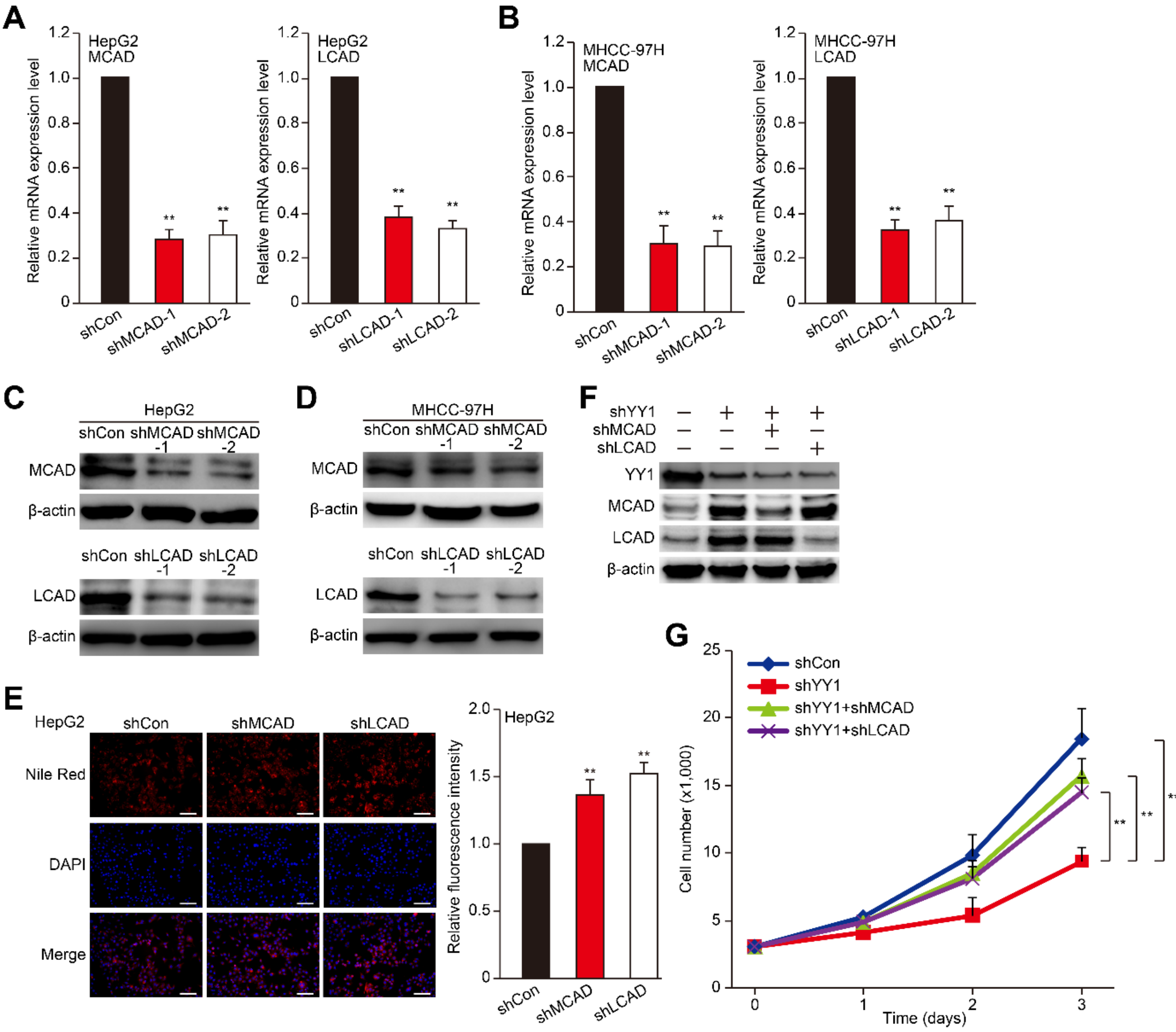

Figure S3. YY1 regulates HCC cell proliferation by inhibiting the expression of MCAD and LCAD. A–B. MCAD and LCAD mRNA expression levels in *MCAD*- or *LCAD*-silenced HepG2 (A) and MHCC-97H (B) cells, as determined using qRT-PCR (n = 3). **C–D.** MCAD and LCAD protein expression levels in *MCAD*- or *LCAD*-silenced HepG2 (C) and MHCC-97H (D) cells, as determined using western blotting. **E.** The accumulation of lipid droplets in *MCAD*- or *LCAD*-silenced HepG2 cells, as determined using Nile Red staining. Scale bars: 200 μm. Representative images (left) and relative fluorescence intensity (right) are shown (n = 9). **F.** YY1, MCAD and LCAD protein expression levels in *YY1/MCAD*- and *YY1/LCAD*-double silenced HepG2 cells, as determined using western blotting. **G.** The number of *YY1/MCAD*- and *YY1/LCAD*-double silenced HepG2 cells at indicated time points. All experiments were done under hypoxic condition. Cells transfected with control vector (shCon) were used as controls. β-actin was used for qRT-PCR normalization and as western blotting loading control. Quantification data are shown as mean ± SEM of three independent experiments. ***P* < 0.01 (ANOVA).

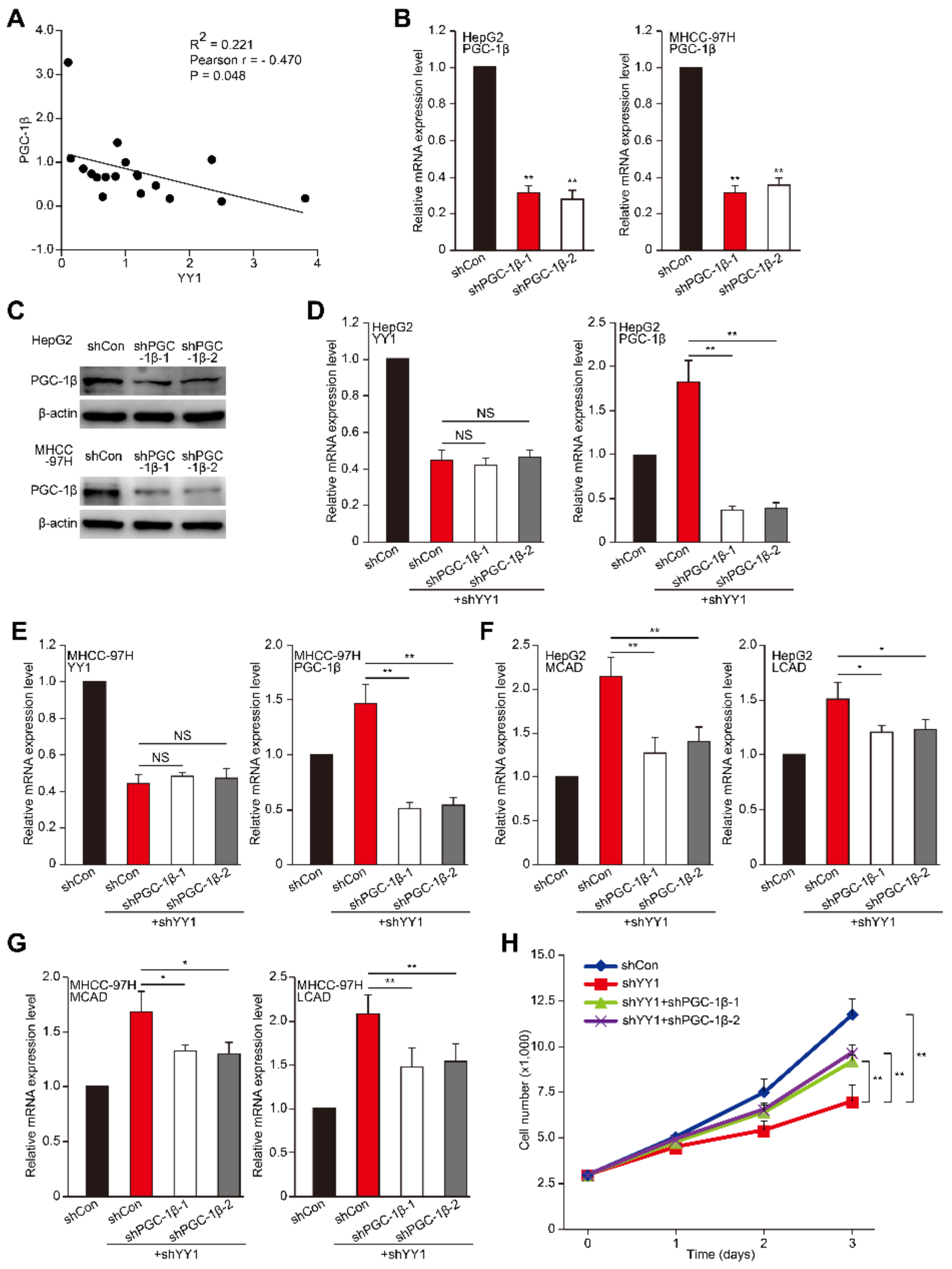

A
R^2 = 0.221
Pearson r = - 0.470
P = 0.048
PGC-1β
YY1
B
HepG2
PGC-1β
MHCC-97H
PGC-1β
Relative mRNA expression level
shCon
shPGC-1β-1
shPGC-1β-2
C
HepG2
shCon
shPGC -1β-1
shPGC -1β-2
PGC-1β
β-actin
MHCC -97H
D
HepG2
YY1
HepG2
PGC-1β
NS
+shYY1
E
MHCC-97H
YY1
MHCC-97H
PGC-1β
F
HepG2
MCAD
HepG2
LCAD
G
MHCC-97H
MCAD
MHCC-97H
LCAD
H
shCon
shYY1
shYY1+shPGC-1β-1
shYY1+shPGC-1β-2
Cell number (x1,000)
Time (days)

Figure S4. YY1 regulates HCC cell proliferation by inhibiting the expression of PGC-1β. A. Correlation analysis between the mRNA expression levels of YY1 and PGC-1β in normal adjacent tissue (n = 18). **B.** PGC-1β mRNA expression level in *PGC-1β*-silenced HepG2 (left) and MHCC-97H (right) cells, as determined using qRT-PCR (n = 3). **C.** PGC-1β protein expression level in *PGC-1β*-silenced HepG2 (top) and MHCC-97H (bottom) cells, as determined using western blotting. **D–E.** YY1 (left) and PGC-1β (right) mRNA expression levels in *YY1/PGC-1β*-double silenced HepG2 (D) and MHCC-97H (E) cells, as determined using qRT-PCR (n = 3). **F–G.** MCAD (left) and LCAD (right) mRNA expression levels in *YY1/PGC-1β*-double silenced HepG2 (F) and MHCC-97H (G) cells, as determined using qRT-PCR (n = 3). **H.** The number of *YY1/PGC-1β*-double silenced HepG2 cells at indicated time points. All experiments were done under hypoxic condition. Cells transfected with shCon were used as controls. β-actin was used for qRT-PCR normalization and as western blotting loading control. Quantification data are shown as mean ± SEM of three independent experiments. *$P < 0.05$; **$P < 0.01$; NS: not significant (ANOVA).

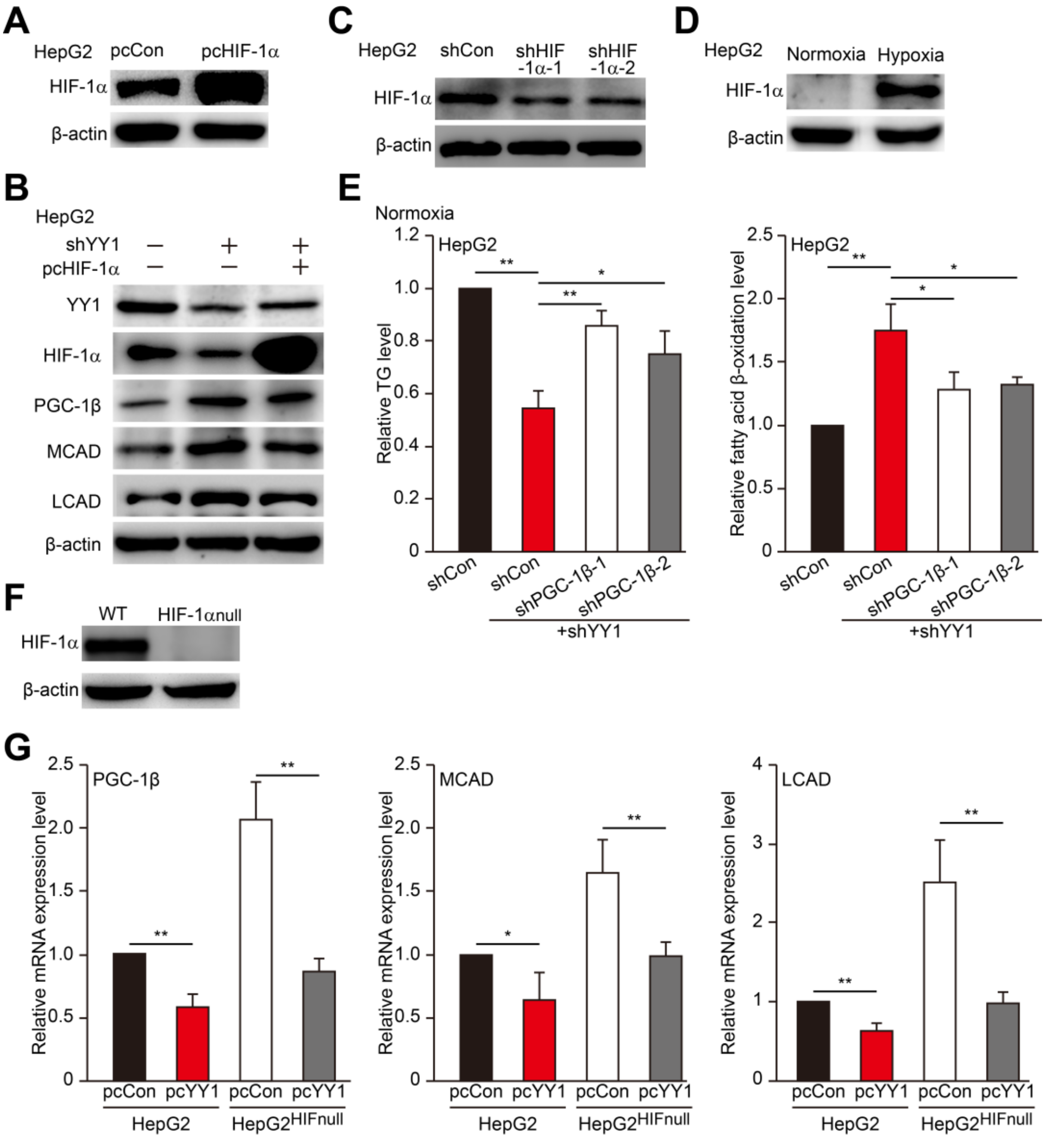

Figure S5. YY1 regulates PGC-1β under normoxic condition. A. HIF-1α protein expression level in *HIF-1α*-overexpressed HepG2 cells cultured under hypoxic condition. **B.** PGC-1β, MCAD and LCAD protein expression levels in *YY1*-silenced, *HIF-1α*-overexpressed HepG2 cells cultured under hypoxic condition. **C.** HIF-1α protein expression level in *HIF-1α*-silenced HepG2 cells cultured under hypoxic condition. **D.** HIF-1α protein expression level in HepG2 cells cultured under normoxic or hypoxic condition. **E.** The level of cellular TG (left) and fatty acid β-oxidation (right) in *YY1/PGC-1β*-double silenced HepG2 cells cultured under normoxic condition (n = 3). **F.** HIF-1α protein expression level in HepG2^{HIFnull} cells cultured under hypoxic condition. **G.** PGC-1β, MCAD, and LCAD mRNA expression levels in HepG2^{HIFnull} cells overexpressing YY1 cultured under hypoxic condition, as determined using qRT-PCR (n = 3). Cells transfected with control vector (shCon or pcCon) were used as controls. Protein expression levels were determined by western blotting. β-actin was used for qRT-PCR normalization and as western blotting loading control. Quantification data are shown as mean ± SEM of three independent experiments. pcCon: pcDNA3.1(+); $*P < 0.05$; $**P < 0.01$ (ANOVA).

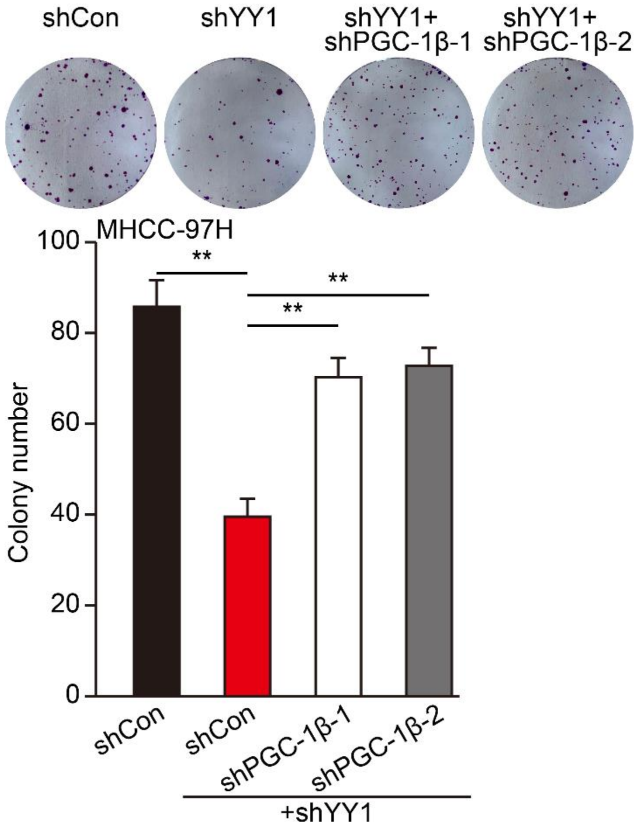

Figure S6. YY1 promotes HCC cells colony formation potential under normoxic condition by regulating PGC-1β. Colony formation potential of *YY1/PGC-1β*-double silenced MHCC-97H cells cultured under normoxia. Representative images (top) and quantification results (bottom, n = 6) are shown. Cells transfected with control vector (shCon) were used as controls.

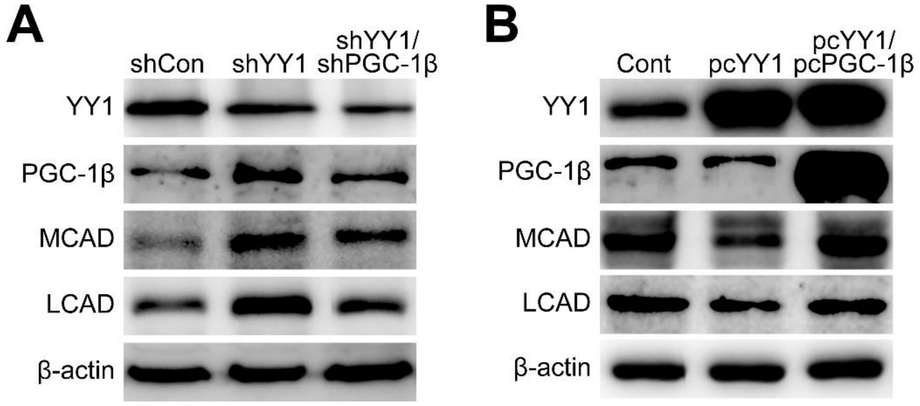

Figure S7. Establishment of *YY1/PGC-1β*-double silenced and *YY1/PGC-1β*-double overexpressed MHCC-97H stable cell lines. A. YY1, PGC-1β, MCAD and LCAD protein expression levels in *YY1*-silenced (shYY1) and *YY1*/*PGC-1β*-double silenced (shYY1/shPGC-1β) MHCC-97H stable cell lines, as determined using western blotting. **B.** YY1, PGC-1β, MCAD and LCAD protein expression levels in *YY1*-overexpressed (pcYY1) and *YY1*/*PGC-1β*-double overexpressed (pcYY1/pcPGC-1β) MHCC-97H stable cell lines, as determined using western blotting. Cells transfected with control vector (shCon or Cont) were used as controls. Cont: pcEF9-puro. β-actin was used as western blotting loading control.

Table S1

Primer pairs used for gene quantification by quantitative RT-PCR.

**Genes**	**Refseq No.**	**Forward primer sequence(5'-3')**	**Reverse primer sequence(5'-3')**
YY1	NM_003403.4	GCGGAGCCCTCAGCCATGGCCTCG	CAGCGGCTGCAGAGCGATCATGG
HMGCR	NM_001130996.1	AAGAAGACAGCCTGAATAG	ATCCTCCACAAGACAATG
PECR	NM_018441	AACCTACCTCCCACAAAG	ATTATTCACCTCCTCCTCATT
FABP3	NM_004102.4	GAGTGGGCAGAAATAACG	ATTAACAGGCTCCGAGAC
LPIN1	NM_001349208.1	TTTCCACGTCCGCTTTGGG	GTGGCCAGGTGCATAGGG
FASN	NM_004104.4	AAGGACCTGTCTAGGTTTGATGC	TGGCTTCATAGGTGACTTCCA
CPT1A	NM_001876.3	ATCAATCGGACTCTGGAAACGG	TCAGGGAGTAGCGCATGGT
CPT1B	NM_152246.2	CCTGCTACATGGCAACTGCTA	AGAGGTGCCCAATGATGGGA
ACSL1	NM_001995	CGACGAGCCCTTGGTGTATTT	GGTTTCCGAGAGCCTAAACAA
ACSL5	NM_203380	CTCAACCCGTCTTACCTCTTCT	GCAGCAACTTGTTAGGTCATTG
ACSL6	NM_001205251	CGCTACATCATCAATACAGCGG	GCATGGACTTAATGACCACCC
SBCAD	NM_001609	GATGGCAAATGTAGACCCTACC	AAGGCCCGGAGTATCACGA
SCAD	NM_000017	CGGCAGTTACACACCATCTAC	GCAATGGGAAACAACTCCTTCTC
MCAD	NM_001127328	GGAAGCAGATACCCCAGGAAT	AGCTCCGTCACCAATTAAAACAT
LCAD	NM_001608	TGCAATAGCAATGACAGAGCC	CGCAACTACAATCACAACATCAC
PPARγ	NM_138711.3	TTGCTGTCATTATTCTCAGTGGA	GAGGACTCAGGGTGGTTCAG
SREBP	NM_004176.4	CTGGTCTACCATAAGCTGCAC	GACTGGTCTTCACTCTCAATG
PGC-1β	NM_001172699	CAGACAGAACGCCAAGCATC	TCGCACTCCTCAATCTCACC
β-actin	NM_001101.3	CGAGCGCGGCTACAGCTT	TCCTTAATGTCACGCACGATTT

Table S2

Antibodies used for western blotting, immunohistochemical staining and ChIP assay.

**Antibody**	**Product number**	**Maker**	**Experiment**	**Dilution**
anti-YY1	#sc-1703	Santa Cruz Biotechnology	Western blotting<br>Immunohistochemistry<br>Chromatin Immunoprecipitation	1/3000<br>1/200<br>30 μg/ml cell lysate
anti-YY2	Ab-116507	Abcam	Western blotting	1/500
anti-MCAD	55210-1-AP	Proteintech	Western blotting<br>Immunohistochemistry	1/1000<br>1/500
anti-LCAD	17256-1-AP	Proteintech	Western blotting<br>Immunohistochemistry	1/1000<br>1/500
anti-PGC-1β	sc-67285	Santa Cruz Biotechnology	Western blotting<br>Immunohistochemistry	1/800<br>1/500
anti-HIF-1α	GTX127309	Gene Tex	Western blotting	1/1000
anti-β-actin	60008-1- Ig	Proteintech	Western blotting	1/50000
anti-Histone H3	17168-1-AP	Proteintech	Chromatin Immunoprecipitation	30 μg/ml cell lysate
anti-Ki67	GB13030-2	Servicebio	Immunohistochemistry	1/500
Goat Anti-Rabbit IgG	ZB2301	ZSGB-BIO	Western blotting	1/10000
Goat Anti-Mouse IgG	ZB2305	ZSGB-BIO	Western blotting	1/10000
DAPI	C1006	Beyotime	Immunofluorescence	not diluted
